# Environmental Factors Affecting Spatio-Temporal Distribution of Crop-Exploiting Species: Implications for Coexistence Between Agricultural Production and Avifauna Conservation in Wetlands

**DOI:** 10.1007/s00267-024-02028-7

**Published:** 2024-08-04

**Authors:** Thazin Htay, Kyaw Kyaw Htoo, Eivin Røskaft, Thor Harald Ringsby, Peter Sjolte Ranke

**Affiliations:** 1 Department of Biology, Norwegian University of Science and Technology (NTNU), Trondheim, Norway; 2 Nature and Wildlife Conservation Division, Forest Department, Ministry of Natural Resources and Environmental Conservation, Nay Pyi Taw, Myanmar; 3 Division of Forest and Biomaterials Science, Graduate School of Agriculture, Kyoto University, Kyoto, Japan; 4 Centre for Biodiversity Dynamics (CBD), Department of Biology, Norwegian University of Science and Technology (NTNU), Trondheim, Norway; 5 BirdLife Norway, Trondheim, Norway

**Keywords:** Bird and agriculture interaction, Bird conservation, Crop-exploiting species, Human–wildlife conflicts and coexistence, MaxEnt, Species distribution modeling

## Abstract

Bird communities in agroecosystems bring both ecosystem services (e.g., pollination) and disservices (e.g., crop exploitation) to farmers. However, in the proximity of wetland reserves, farmers disproportionately experience harvest yield loss due to large aggregation of bird species that can utilize various agricultural resources. This often results in negative human–wildlife interactions which lower conservation support among farmers. Knowledge about the distribution of avian species that negatively influence yields, and its environmental drivers is thus fundamental to reconcile crop production and bird conservation. This study aims to examine the spatio-temporal patterns in richness and abundance of bird species known to cause agricultural yield loss as well as species-specific distribution patterns for the six bird species that are most challenging for local farmers. In combination with interview surveys of local farmers (*n* = 367) and seasonal bird surveys (*n* = 720), we investigated distribution of crop-exploiting avian species in the Indawgyi wetland ecosystem in Myanmar. Our results showed high richness and abundance of crop-exploiting species in the water habitat across all seasons, with most challenging species exhibiting higher presence closer to these water sources. The crop phenology had positive effect on species richness and abundance during the growing season. The agricultural use of crop-exploiting species was season- and species-specific, where the presence probability in the agricultural habitat was higher in habitat generalists than wetland specialists. Therefore, we suggest improved management of natural wetland habitats (e.g., habitat restoration), sustainable coexistence mechanisms in farms close to water (e.g., bird-friendly rice farming and Ecolabel certification) to reduce avian impacts on the farming communities and, at the same time, to promote bird conservation in wetlands of international importance.

## Introduction

Wetlands are the most productive ecosystems of the world and are home to about 40% of all plant and animal species (Convention on Wetlands 2021). Birds are the most well-known group of species among the wetland biota and thus function as wetland indicators. Globally, wetlands serve as primary habitats for migratory birds and a quarter of all wetland-dependent bird species are facing risks of extinction (Şekercioğlu et al. 2023). However, wetlands are closely associated with anthropogenic landscapes and their biological integrity is affected by various human impacts (Fluet-Chouinard et al. 2023; IPBES 2019). Between 1970 and 2015, one-third of global wetlands have been lost (Davidson 2018), mainly due to agricultural expansion (Gell et al. 2023). Wetland deterioration has resulted in the reduction in extent and quality of important bird habitats and the widespread decline of wetland-dependent birds (Davidson 2018; IPBES 2019). Wetlands International (2012) reported a decline in 38% of known waterbird populations. In the face of rapid changes in land uses and climate, finding a sustainable solution that can fulfill the resource demand for growing human population without compromising the ecological value of wetlands has become a major challenge (Blount et al. 2021; Gell et al. 2023; Nilsson et al. 2018). While conservation of natural habitats through well-managed protected area system is of primary importance, restoration of degraded habitats and integration of its surrounding landscapes through adoption of socio-ecologically sustainable practices is also crucial to preserve wetland bird habitats in the future (IPBES 2019; Kleijn et al. 2020; Ma et al. 2010; Munguía and Heinen 2021; Smart et al. 2014; Wright et al. 2012; Yong et al. 2022).

Agroecosystems adjacent to wetlands are often favorable bird habitats if managed sustainably (Blount et al. 2021). Although agricultural habitats cannot fully replace natural habitats, several species benefit from wetland-associated cultivated landscapes (Ma et al. 2010; Natuhara 2013; Parejo et al. 2019), mostly waterbirds including species of major conservation concern (Glisson et al. 2017; Sundar and Kittur 2013). While the presence of bird species may benefit agriculture through pollination and pest control, the use of agricultural fields by birds may also be accompanied with yield loss due to herbivory, foraging, trampling, and puddling (Fox et al. 2017; Huang et al. 2023; Klug et al. 2023). Examples include herbivorous waterfowl (Fox et al. 2017; Nilsson et al. 2018), doves and parakeets (Codesido et al. 2015), and passerines such as sparrows, weavers and munias (Angkaew et al. 2022). Bird species’ exploitation of commercially important crops (mostly cereals and oil crops) impose socio-economic costs to the farmers and provoke negative attitudes toward birds (Canavelli et al. 2013; Htay et al. 2022; McMahon et al. 2024). In serious socio-economic losses, this has even led to illegal killing of birds (Htay et al. 2023a). Recent meta-analyses on bird and agricultural interaction revealed that economic vulnerability and negative perceptions toward birds were especially high among farmers of the less-developed countries due to the absence of responsible management strategies (Araneda et al. 2022; Huang et al. 2023; Klug et al. 2023). Consequently, these farmers are reluctant to support conservation of birds and bird habitats and the adoption of bird-friendly agricultural practices (Nilsson et al. 2018). Therefore, addressing biodiversity challenges in agriculture-dominated landscapes requires transformative changes that recognize and reconcile diverse interests and values of primary stakeholders (IPBES 2019). Conservation management that promotes coexistence between wildlife preservation and crop production is crucial for maintaining viable bird populations and minimizing crop losses for farmers. This can be achieved through socio-ecologically sound practices (IPBES 2019; McMahon et al. 2024).

Avian impacts on agriculture are of conservation concern in the wetlands located along the major bird migratory flyways (Angkaew et al. 2022; Montràs‐Janer et al. 2019; Nilsson et al. 2019). As most studies on the topic have been conducted in Europe and America, the issue has mostly been overlooked in Asia so far (Fox et al. 2017). A recent study of the East Asian Australasian Flyway revealed that netting and trapping to exclude crop-exploiting birds also kill several of the non-targeted birds including migratory species (Angkaew et al. 2022; Yong et al. 2022; Htay et al. 2023a). The challenge is also prevalent in Myanmar where more than half of the country’s wetland areas are human created (mostly rice fields) and are linked with natural wetland habitats (Forest Department 2018). A previous study in an agroecosystem surrounding Myanmar’s largest freshwater lake reported that 87.5% of farming communities experienced avian crop damage and the severity reached up to 75% of single farm (Htay et al. 2022). Avian damages on agricultural crop and negative attitudes toward the species, in combination with the lack of appropriate mitigation measures have led to various forms of illegal bird killing in this landscape (Htay et al. 2023a). These findings call for effective management tools and mitigations to reduce negative impacts on avian population dynamics as well as farmers’ agricultural production (Glikman et al. 2021; Huang et al. 2023). To implement effective mitigation and coexistence measures, it is first necessary to acquire knowledge in order to identify which ecological factors influence the distribution of crop-utilizing avian species and determine where and when management actions should be prioritized (McMahon et al. 2024).

Until now, studies have mainly focused on habitat-centered approaches and demonstrated that farm characteristics (e.g., farm size, farm shape, crop type, and vegetation heterogeneity) and its proximity to major bird habitats are the main factors influencing avian use of agricultural habitats (Calamari et al. 2018; Canavelli et al. 2014). However, the distribution of bird species varies across space and time in response to favorable climate and aggregated resources within the landscape (Amano et al. 2008; Canavelli et al. 2014; Codesido et al. 2015; Girma et al. 2017; Zufiaurre et al. 2016). In this regard, the abundance of birds in agricultural habitats cannot be solely attributed to resources within such areas; rather, it is influenced by the availability of neighboring habitat’s resource abundance (Calamari et al. 2018; Zufiaurre et al. 2017). Additionally, variation in species metabolic requirements between breeding and non-breeding periods influence their space use and interaction with the environment (McPherson and Jetz 2007). This distributional arrangement may further intertwin with the species’ life-history traits such as habitat preferences, dietary breadth, foraging behaviors, efficiency in gathering food resources, and tolerance to anthropogenic disturbances (Araneda et al. 2022; Briceño et al. 2019; Glisson et al. 2017; Sica et al. 2020). In tropical agroecosystems, there is substantial seasonality and changes in temperature and precipitation regimes, microclimatic conditions, landscape structures, agricultural practices and crop phenology have profound influences on the spatial and temporal distribution of birds (Girma et al. 2017; Sausse et al. 2021; Studholme et al. 2018; Tozer et al. 2010). Therefore, in addition to a habitat-centered approach, a landscape-centered approach accounting for spatial and temporal dynamics is necessary to comprehensively understand the underlying ecological factors influencing the distribution of crop-exploiting avian species, which is fundamental to develop sustainable habitat management strategies (Sausse et al. 2021).

With this study, we aimed to examine the spatio-temporal variation in the diversity crop-exploiting bird species and its ecological drivers in the Indawgyi wetland ecosystem in Myanmar using both approaches; at the landscape level (A), we asked; (1) how habitat types, topographic features, climatic conditions, and anthropogenic factors influence the diversity of bird species known to cause crop losses, (2) how diversity of species that reduced crop yields correlates with other species in the bird community, and (3) how six species (Purple swamphen *Porphyrio porphyrio*, Lesser whistling duck *Dendrocygna javanica*, Spotted dove *Spilopelia chinensis*, Eurasian tree sparrow *Passer montanus*, Scaly-breasted munia *Lonchura punctulate* and Baya weaver *Ploceus philippinus*) that cause yield loss in more than 10% of the farms, are distributed within the wetland landscape and which environmental factors influence their distributions. At the agricultural habitat level (B), we investigated how the species use of agricultural fields was influenced by the phenology of the agricultural crops and environmental conditions of the farm. Understanding spatial-temporal dynamics of crop-exploiting species and how they interact with landscape elements will provide useful insights needed to develop efficient management strategies that can reduce avian impacts on the farming communities and, at the same time, promote conservation of bird populations in wetlands.

## Methods

### Study Area

The Indawgyi wetland ecosystem is located in the northern part of Myanmar and covers an area ~47,885 ha (Convention on Wetlands 2016). The study area belongs to the subtropical climate with the lowest temperature of 5.5 °C (in January) while the highest is 39 °C (in April). The annual rainfall ranges from 1048 to 2060 mm regularly peaking in July and August (Forest Department 2018). The landscape comprises a natural lake nested within grasslands and riparian forests, and agricultural lands in the outer most part. The unique combination of different landscape elements attracts diverse groups of birds, and the wetland annually receives thousands of migratory birds (Convention on Wetlands 2016). A total of 312 species have been recorded in the study area and several of them are threatened species. Therefore, the landscape is internationally important for avifauna conservation and recognized as an IBA, Ramsar site and UNESCO Biosphere reserve (Forest Department 2018). In this ecosystem, the dynamics of the wetland and its surrounding agricultural landscape are shaped by a seasonal water regime (Forest Department 2018). Crop cultivation is rainfall dependent, and rice is the major crop grown over the region. The agricultural system is characterized by single cropping in most parts of the area. Some villages in the outflow alluvial areas in the northern and eastern parts of the wetland practice double cropping (Htay et al. 2022). Farmers start crop growing with the arrival of rain during the monsoon season through mid-May to September. The lake also receives water in this season. When the waterbody gradually expands, the grassland and riparian forests are inundated until winter (Forest Department 2018). The winter season is from October to mid-February, and the crops are harvested during this period. The summer season lasts from late February to mid-May when most of the crop fields are harvested and fallowed. With intensification and expansion of agriculture, along with fertilizer inputs and sedimentation into the lake, avifauna of the Indawgyi is under different pressures of habitat shrinkage and degradation (Convention on Wetlands 2016). Furthermore, illegal killing is threatening local bird populations due to conflicts between bird conservation and crop production (Htay et al. 2023a).

### Identification of Bird Species That Reduce Agricultural Yield

Bird species that reduced crop yield were identified according to Htay et al. (2022). In consultation with 367 local farmers, they identified a total of 20 species and 6 genera known to cause agricultural yield loss (Htay et al. 2022). The most common species that caused crop damages in more than 10% of the farms were Purple swamphen, Lesser whistling duck, Spotted dove, Eurasian tree sparrow, Scaly-breasted munia, and Baya weaver (see Htay et al. 2022 for methodology and species description details).

### Species Data

Species data were collected from June 2021 to March 2022 in 120 sample plots using the point count method. Following, Ralph et al. (1995), we adopted stratified random sampling, where 30 sample plots were allocated in each of the four habitat types constituted in the landscape (i.e., agricultural land, riparian forest, grassland, and water) (Fig. S2). Sampling points were randomly distributed in ArcMap Desktop using *“Create random points tool”* based on the latest available landcover map (Forest Department 2018) and separated 200 m from each other to reduce spatial bias. The adequacy of our sampling effort was evaluated using sampling completeness curves (Chao and Jost 2012) and the sample size was sufficiently large to detect 95% of species in all habitats (see details in Htay et al. 2023b). As the landcover map that we used for sample plot allocation was developed in 2015, we carried out ground truth surveys to verify the different habitat types. Bird surveys were conducted seasonally, and the sampling points were visited two times in each season: growing season (sampled in June and July 2021), harvest season (sampled in October and November 2021) and after crop harvest season (sampled in February and March 2022). Therefore, each sample plot was visited six times throughout the study and the total number of observations was 720 (i.e., 3 seasons × 2 visits × 120 sample plots). During a time period of 15 min, we recorded all bird species identified either based on sound or observations within 50 m radius of the sampling points. We then divided the data into two datasets specific to: (1) bird species that were identified to reduce agricultural yield, and (2) bird species that caused no agricultural yield loss. The diversity (i.e., richness and abundance) of species belonging to the two groups were calculated for each sample plot using the *Vegan* package (Oksanen et al. 2024).

To prepare species occurrence data for the six species that caused serious yield loss, bird survey data were subset to include only those species. After that, we extracted the location of sampling points where each species was present at least once in each season and created seasonal species occurrence datasets (Glisson et al. 2017; Williams et al. 2017). To minimize sample bias caused by spatial autocorrelation, we calculated Moran I statistics and applied spatial filtering as suggested by Boria et al. (2014) and Kramer‐Schadt et al. (2013). Occurrence points were filtered to include only one presence point within 400 m. As there was no spatial autocorrelation in spatially filtered occurrence records, we used them for seasonal species distribution modeling (Table 1). All spatial analyses were conducted using *spdep* and *spThin* packages in R.

**Table 1:** The number of species occurrence points used in the MaxEnt modeling for the six species that caused most reduction in crop yields

Species	Season	Occurrence points	Moran I	*P*	AUC	TSS
Purple swamphen	Growing season	40 (52)	−0.148	0.966	0.919 (0.039)	0.733 (0.208)
*Porphyrio porphyrio*	Harvest season	21 (29)	−0.197	0.996	0.932 (0.024)	0.668 (0.336)
	After harvest season	23	−0.055	0.712	0.939 (0.007)	0.762 (0.152)
Lesser whistling duck	Growing season	64 (84)	−0.268	0.998	0.893 (0.043)	0.598 (0.137)
*Dendrocygna javanica*	Harvest season	31	0.059	0.202	0.942 (0.023)	0.686 (0.202)
	After harvest season	50	0.005	0.455	0.896 (0.017)	0.737 (0.052)
Spotted dove	Growing season	40 (47)	−0.125	0.93	0.900 (0.030)	0.653 (0.098)
*Spilopelia chinensis*	Harvest season	44 (58)	−0.105	0.909	0.892 (0.018)	0. 659 (0.061)
	After harvest season	35 (43)	−0.074	0.800	0.903 (0.089)	0.684 (0.174)
Eurasian tree sparrow	Growing season	31	0.125	0.053	0.844 (0.050)	0.584 (0.079)
*Passer montanus*	Harvest season	10	0.028	0.255	0.762 (0.186)	0.591 (0.182)
	After harvest season	18	0.073	0.168	0.866 (0.092)	0.573 (0.166)
Scaly-breasted munia	Growing season	17	0.049	0.225	0.841 (0.119)	0.565 (0.336)
*Lonchura punctulata*	Harvest season	14	0.107	0.098	0.700 (0.173)	0.587 (0.227)
	After harvest season	9	0.010	0.246	–	–
Baya weaver	Growing season	88	−0.038	0.674	0.887 (0.048)	0.603 (0.125)
*Ploceus philippinus*	Harvest season	16	0.042	0.227	0.767 (0.095)	0.521 (0.227)
	After harvest season	5	−0.011	0.679	–	–

Number in the parenthesis indicates spatial-unfiltered occurrence points. Moran I and *P* values indicate the results of spatial autocorrelation in the spatially filtered records. AUC and TSS values indicate the accuracy of the model fitted with the spatially filtered occurrence points. See section “Methods” for further information

### Environmental Data

At the landscape level, we used a candidate set of 25 predictors characterizing habitat, topographic, climatic, and anthropogenic effects to parameterize our species distribution models (Bradie and Leung 2017; Ding et al. 2006; Seoane et al. 2004) (Table 2). In our study area, the latest available landcover data developed in 2015 was not consistent with our species occurrence data. Thus, we conducted an updated landcover analysis, following the same classification categories (Forest Department 2018) (see Supplementary Materials for details on landcover classification). Topographic variables including elevation, aspect, and slope were derived from the Digital Elevation Model (DEM) with a 30 m spatial resolution that was downloaded from the NASA Shuttle Radar Topography Mission (SRTM) database (Farr et al. 2007). Bioclimatic variables at a spatial resolution of 30 arc seconds (~1 km around equator) were obtained from the WorldClim database, which includes bioclimatic predictor layers summarizing annual trends, seasonality and extremes in temperature and precipitation (Fick and Hijmans 2017). Distance to water was measured as Euclidean distance to the nearest source of water and calculated from the lake and stream vector layer provided by the Myanmar Forest Department. Distance to village was calculated from the village distribution data provided by the park administration office (Forest Department 2018). All environmental variables were resampled to a 30 m spatial resolution with bilinear interpolation and reprojected to the WGS 1984 coordinate system. We tested correlation among the predictors to avoid multicollinearity (Fig. S3) and included the variables that had correlation <0.7 (Dormann et al. 2013). This resulted in seven correlated groups (Fig. S4) and we keep only one relevant predictor from each group (Barbet-Massin and Jetz 2014). Ultimately, eight variables (habitat type, aspect in terms of northness and eastness, slope, temperature seasonality, precipitation seasonality, distance to water and distance to village) were retained for further analyses (Fig. S5). We ensured that all our selected environmental variables had VIF < 3. All data preparations were conducted in ArcMap v.10.8.2 and R using packages *raster*, *sp*, *spdep*, *ENMTools* and *usdm*.

**Table 2:** Candidate set of habitats, topographic, bioclimatic, and anthropogenic variables used as input variables in the species distribution modeling

Source	Category	Variable	Unit	Resolution
Landcover Map (2022) (supplementary material)	Habitat variables	Habitat	Dimensionless	30 m
SRTM (Farr et al. 2007)	Topographic variables	Elevation	m	30 m
		Slope	Degrees	30 m
		Northness Cos (aspect)	Dimensionless (−1 to +1)	30 m
		Eastness Sin (aspect)	Dimensionless (−1 to +1)	30 m
Forest Department, Myanmar (Forest Department 2018)		Distance to water	m	30 m
WorldClim https://www.worldclim.org/ (Fick and Hijmans 2017)	Bioclimatic variables	Annual mean temperature	°C	~1 km
		Mean diurnal range (mean of monthly (max temp–min temp))	°C	~1 km
		Isothermality (mean diurnal range/temperature annual range) (×100)	%	~1 km
		Temperature seasonality (standard deviation × 100)	°C	~1 km
		Maximum temperature of the warmest month	°C	~1 km
		Min temperature of coldest month	°C	~1 km
		Temperature annual range (max temp of warmest month–min temp of coldest month)	°C	~1 km
		Mean temperature of wettest quarter	°C	~1 km
		Mean temperature of driest quarter	°C	~1 km
		Mean temperature of warmest quarter	°C	~1 km
		Mean temperature of coldest quarter	°C	~1 km
		Annual precipitation	mm	~1 km
		Precipitation of wettest month	mm	~1 km
		Precipitation of driest month	mm	~1 km
		Precipitation seasonality (coefficient of variation)	%	~1 km
		Precipitation of wettest quarter	mm	~1 km
		Precipitation of driest quarter	mm	~1 km
		Precipitation of warmest quarter	mm	~1 km
		Precipitation of coldest quarter	mm	~1 km
Forest Department, Myanmar (Forest Department 2018)	Anthropogenic variables	Distance to village	m	30 m

At the agricultural habitat level, we recorded phenology (i.e., growth stages) of the cultivated crop during our bird surveys. In the crop growing season, only rice is cultivated, and the stages were categorized as field plowing, rice seed broadcasting, germinating, and growing. The crop stages in the harvest season included growing, flowering, ripening, and harvesting. After the harvest season, most rice fields are fallowed or prepared for the next rice growing season. Only the fields in the northern and eastern parts grow second crops (beans, peanuts, and mustard). So, the stages in this season included harvested rice, growing soybean, growing mustard, fired stubble, and fallow.

### Statistical Analyses

#### Landscape Level

We fitted generalized linear mixed effect models (GLMMs) to examine seasonal variation in the diversity (i.e., richness and abundance, respectively) of crop-exploiting bird species and their association with environmental factors at the landscape level. We constructed the models for each of the three seasons separately by constructing models for richness and abundance of crop-exploiting birds as response variables, and environmental variables (habitat, slope, northness, eastness, temperature seasonality, precipitation seasonality, distance to water and distance to village) as predictors (*n* = 240). We did not account for detection probability as our sampling points were visited only two times in each season and did not fulfill the required statistical assumption for imperfect detection (MacKenzie and Royle 2005). Therefore, the identity of sample plots and sampling occasions was included as random factors to account for non-independence within sampling sites and sampling visits. All covariates were mean centered and scaled. The model for species richness was fitted using poisson distribution and the model for species abundance was fitted using negative binomial distribution due to overdispersion. A variable was considered to have significant effect if the confidence interval of the estimate did not include zero. The significant effects of predictor variables observed in the GLMM models were also evaluated using likelihood ratio tests (LRT) by assessing the goodness of fit of the full model with the models that excluded a specific predictor successively (Lewis et al. 2011). As there was no opposing effect between GLMM and LRT (Tables S2–5), we reported the results from the GLMM. All models were run using *glmmTMB* package (Brooks et al. 2007) and the residual diagnostics of the models were checked using the *DHARMa* package (Hartig 2022). Spatial autocorrelation of the model residuals was evaluated using Moran I statistics under *spdep* package (Bivand 2022). No significant autocorrelation was detected in our fitted model (Table S6). We carried out a Pearson correlation analysis to examine how seasonal richness and abundance of crop-exploiting species correlated with other species within the wetland landscape. As our sampling points were visited two times in each season, we used the maximum richness and abundance for each sample point to avoid temporal pseudo replication (Johnson 2008).

We used maximum entropy modeling (MaxEnt) to predict the seasonal distribution of the six species that caused yield loss in more than 10% of the farms and to examine the influence of environmental factors on species presence (Phillips et al. 2006). We fitted MaxEnt models for each of the six species in each season. Species with <10 occurrences were not included in the analysis (Wisz et al. 2008). Therefore, the distribution of Scaly-breasted munia and Baya weaver after the harvest season was excluded. When developing the model, we randomly allocated 75% of the occurrence records for model training and the remaining 25% for model testing (Fielding and Bell 1997). We then parameterized the models with 10,000 random background points and 7 environmental predictors (habitat, slope, aspect, temperature seasonality, precipitation seasonality, distance to water and distance to village). As MaxEnt model output can be highly sensitive to overfitting in the default model settings (Merow et al. 2013), we carried out a model selection process using *ENMeval* package (Muscarella et al. 2014). To reduce the complexity of the model, and the likelihood of overparameterization, we calibrated the model with different combination of feature types (linear, quadradic and hinge features) and regularization parameters (0.5, 1, 2, 3, 4, following Morales et al. 2017). After that, all models were ranked according to their fit to the data using AICc and choose the most parsimonious model (Burnham and Anderson 2002). We cross validated our selected model with four replicates to increase model performance and used the average model for the subsequent presence area prediction (Fielding and Bell 1997). Model accuracy was assessed using threshold-independent and threshold-dependent approaches (Allouche et al. 2006; Merow et al. 2013). As a threshold-independent evaluation, we used area under the receiver operator curve (AUC) which ranges from 0 (random) to 1 (perfect distinction) (Phillips et al. 2006). Higher AUC values demonstrate models with better distinctive capacity, while AUC values <0.5 indicate models cannot discriminate between preference habitat and environmental background. AUC values >0.7 are considered as models with good performance and suitable for conservation planning (Baldwin 2009). As a threshold-dependent method, we used True Skill Statistics (i.e., sensitivity + specificity − 1) which evaluates the accuracy of predicted model by considering omission and commission error (Liu et al. 2016). TSS was calculated using the maximum sum of sensitivity and specificity. TSS ranges from −1 to +1, and value of 1 indicates perfect model performance, and values ≤ 0 indicate model performance that is no better than random (Allouche et al. 2006; Glisson et al. 2017). We calculated average AUC and TSS of the four cross-validated replications to obtain a more robust estimate. For easy interpretation, we transformed the continuous occurrence probability map into binary map (presence/absence prediction) using the maximum training sensitivity and specificity threshold. Areas with an occurrence probability above the threshold are regarded as predicted presence areas for each species. Although this discretization may lose some information, presenting the information as presence/absence areas may be more practical in conservation decisions and management practice. The contribution of the variables to the model was evaluated by their permutational importance >5% (Table 3) (Rodriguez-Ruiz et al. 2019). MaxEnt modeling was implemented using the *dismo* package (Hijmans et al. 2015).

**Table 3:** Environmental variables used in the species distribution models of most challenging species and their contributions to the models

Variable	Purple swamphen			Lesser whisting duck			Spotted dove			Eurasian tree sparrow			Scaly-breasted munia			Baya weaver		
Seasons	G	H	AH	G	H	AH	G	H	AH	G	H	AH	G	H	AH	G	H	AH
Habitat	**23.4**	**28.6**	**10.8**	**8.2**	**13.9**	**15.1**	**21.2**	**25.7**	**13.2**	**12.5**	2.3	0.1	**12.9**	2.7	**–**	**12.7**	**11**	**–**
Slope	0.4	0.7	0	3.3	0	0	3.2	4	0.2	0	0	0	2.9	2.8	**–**	0.5	0.6	**–**
Northness	0	0	0	0	4.4	4.3	**5.2**	2.8	3.8	0	0	0	0.6	0	**–**	0	0	**–**
Eastness	0.3	0.5	0	1.2	0	0.3	2.5	0.5	0.6	0	0.4	0	0	2.1		0	0	
Temperature seasonality	3.6	**9.9**	0.7	2.4	0.9	0.5	2.9	3	2.7	2.3	0.5	0	0	0	**–**	3.2	3.2	**–**
Precipitation seasonality	0	2.4	0	2.5	0.4	3.5	0	0	0	0.6	0	0	0	0	**–**	0	0	**–**
Distance to water	**65.6**	**55.7**	**86.5**	**65.7**	**73.9**	**61.6**	**40.3**	**40.8**	**63.2**	**11.2**	**11.3**	**5.6**	**49.7**	**20.1**	**–**	**71.2**	**32.1**	**–**
Distance to village	**6.7**	2.2	2	**16.3**	**6.5**	**14.7**	**24.7**	**23.2**	**16.3**	**73.4**	**85.5**	**94.3**	**33.9**	**72.3**	–	**12.4**	**53.1**	–

Most important variables that have variable importance >5%, in terms of contribution to the model in each model are shown in bold

*G* crop growing season, H crop harvest season and AH after harvest season

#### Habitat Level

At the habitat level, we extracted the species data to include only agricultural habitat and fitted GLMMs to evaluate how seasonal variation in crop phenology and environmental conditions of the farms influenced the richness and abundance of crop-exploiting species (*n* = 60). All analyses were performed in R (R Core Team 2022).

## Results

### Spatio-Temporal Variation in the Richness and Abundance of Crop-Exploiting Species

**Fig. 1:**
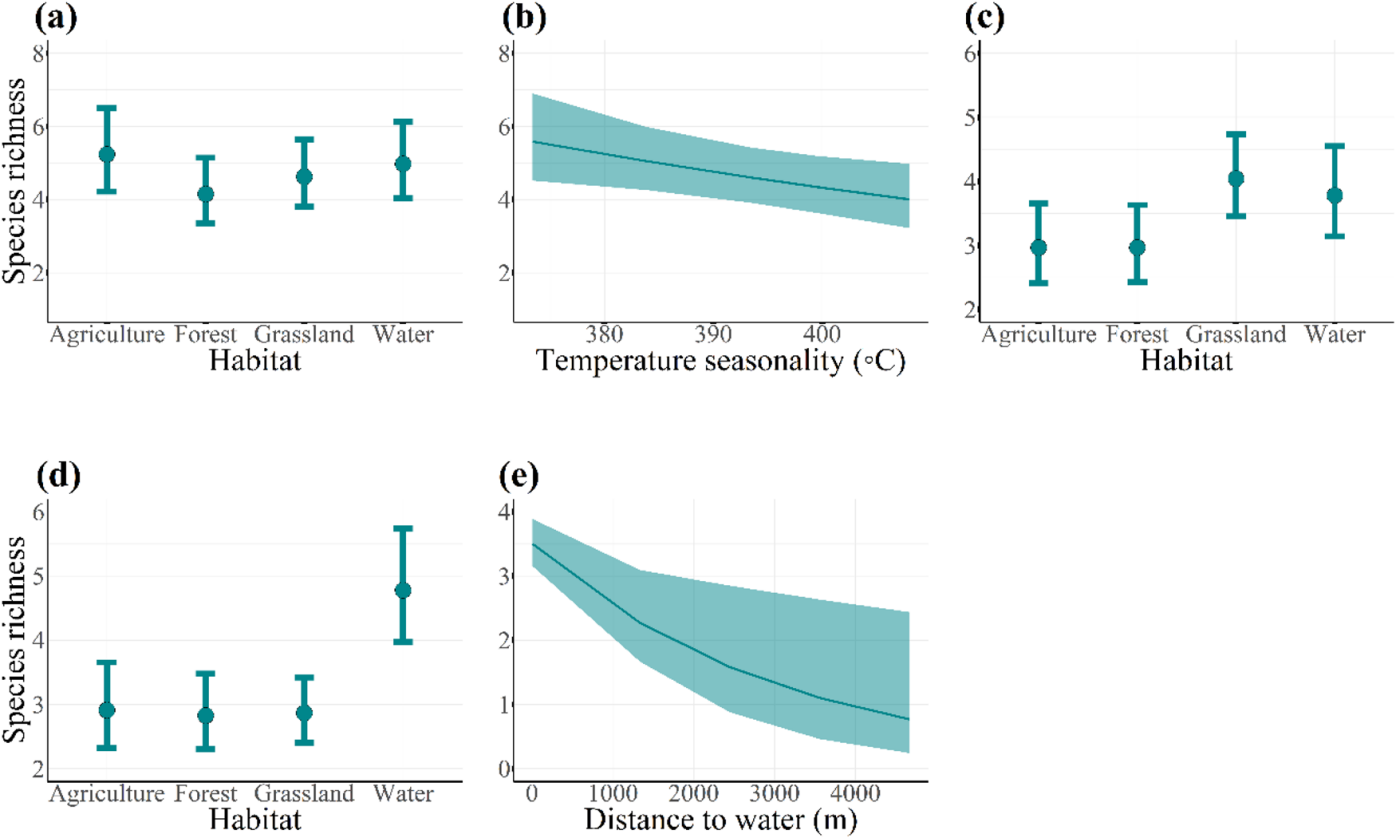
Species richness of crop-exploiting species during the growing season in relation to habitat type and temperature seasonality (**a**, **b**), those of the harvest season in relation habitat type (**c**) and those after harvest season in relation to habitat type and distance to water (**d**, **e**). See Table 4 for parameter estimates and statistics

We found seasonal variation in the effect of environmental factors on the richness and abundance of crop-exploiting bird species (Table 4). Habitat types, temperature seasonality and distance to water were the main factors affecting the species richness (Table 4). In the growing season, species richness was higher in agricultural land, grassland, and water than forest (*β* = −0.232 [−0.457, −0.006], SE = 0.115, Table 4a, Fig. 1a). Temperature variation had negative effect on species richness (*β* = −0.083 [−0.154, −0.012], SE = 0.036, Table 4a, Fig. 1b). During the harvest season, species richness was higher in the grassland (*β* = 0.309 [0.025, 0.583], SE = 0.149, Table 4b, Fig. 1c), water (*β* = 0.242 [0.056, 0.541], SE = 0.123, Table 4b, Fig. 1c). After the harvest season, species richness was the highest in the water habitat (*β* = 0.496 [0.173, 0.818], SE = 0.165, Table 4c, Fig. 1d) and it decreased with increasing distance to water (*β* = −0.181 [−0.323, −0.038], SE = 0.073, Table 4b, Fig. 1e).

**Table 4:** Model predicting effects of environmental characteristics on the richness of crop-exploiting bird species in the Indawgyi wetland ecosystem

Predictor variables	Estimate	SE	CI	
			Lower	Upper
a. Growing season				
**Intercept**	**1.655**	**0.111**	**1.440**	**1.870**
**Habitat (forest)**	−**0.232**	**0.115**	−**0.457**	−**0.006**
Habitat (grassland)	−0.124	0.111	−0.341	0.094
Habitat (water)	−0.050	0.120	−0.286	0.186
Slope	−0.005	0.040	−0.083	0.073
Northness	−0.027	0.039	−0.104	0.050
Eastness	−0.020	0.034	−0.086	0.046
**Temperature seasonality**	−**0.083**	**0.036**	−**0.154**	−**0.012**
Precipitation seasonality	0.037	0.035	−0.032	0.106
Distance to water	0.007	0.038	−0.068	0.082
Distance to village	0.034	0.034	−0.033	0.101
Random variable: Plot ID	SD = 1.843e − 05			
Random variable: sampling occasion	SD = 1.085e − 01			
b. Harvest season				
**Intercept**	**1.087**	**0.106**	**0.878**	**1.297**
Habitat (forest)	0.001	0.151	−0.295	0.295
**Habitat (grassland)**	**0.309**	**0.149**	**0.025**	**0.583**
**Habitat (water)**	**0.242**	**0.123**	**0.056**	**0.541**
Slope	−0.013	0.052	−0.114	0.088
Northness	−0.052	0.049	−0.150	0.044
Eastness	−0.063	0.042	−0.146	0.020
Temperature seasonality	0.069	0.045	−0.020	0.159
Precipitation seasonality	−0.004	0.043	−0.089	0.080
Distance to water	0.044	0.050	−0.044	0.132
Distance to village	0.035	0.042	−0.049	0.118
Random variable: Plot ID	SD = 0.141			
Random variable: sampling occasion	SD = 0.033			
c. After harvest season				
**Intercept**	**1.068**	**0.116**	**0.841**	**1.296**
Habitat (forest)	−0.029	0.159	−0.341	0.283
Habitat (grassland)	−0.014	0.155	−0.318	0.289
**Habitat (water)**	**0.496**	**0.165**	**0.173**	**0.818**
Slope	−0.048	0.053	−0.152	0.055
Northness	0.033	0.053	−0.071	0.137
Eastness	0.040	0.045	−0.048	0.128
Temperature seasonality	0.013	0.046	−0.077	0.103
Precipitation seasonality	−0.062	0.043	−0.147	0.023
**Distance to water**	−**0.181**	**0.073**	−**0.323**	−**0.038**
Distance to village	−0.004	0.043	−0.088	0.079
Random variable: Plot ID	SD = 0.139			
Random variable: sampling occasion	SD = 0.036			

Species richness in the avian community was used as a response variable whereas seven different environmental variables were used as predictors. Environmental variables are standardized with mean zero and a standard deviation. The analysis was based on a generalized linear mixed model fitted with Poisson distribution using glmmTMB (Brooks et al. 2007). The identity of the bird sampling points, and sampling occasion were included as random factors. We present effect size of predictors, their standard errors and 95% confidence intervals, reference category for the habitat is Habitat (Agricultural land). CIs not overlapping zero in bold font

**Fig. 2:**
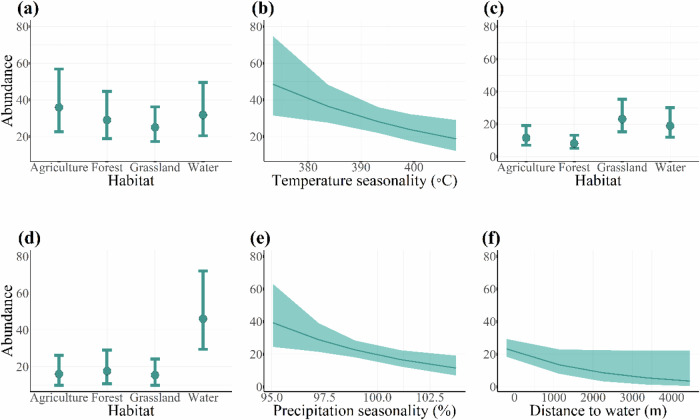
Abundance of crop-exploiting species during the growing season in relation to habitat type, and temperature seasonality (**a**, **b**), those of the harvest season in relation to habitat type (**c**) and those after harvest season in relation to habitat type, precipitation seasonality and distance to water (**d**–**f**). See Table 5 for parameter estimates and statistics

Habitat types, temperature seasonality, precipitation seasonality and distance to water were found as the main factors affecting the abundance of crop-exploiting species (Table 5). In the growing season, species abundance was not different among habitat types (Table 5a, Fig. 2a). However, it was lower in areas with temperature variations (*β* = −0.237 [−0.416, −0.057], SE = 0.092, Table 5a, Fig. 2b). In the harvest season, species abundance was higher in the grassland (*β* = 0.691 [0.026, 1.357], SE = 0.339, Table 5b), water (*β* = 0.493 [0.240, 0.7418], SE = 0.129, Table 5b, Fig. 2c). After the harvest season, species abundance was the highest in water (*β* = 1.052 [0.315, 1.788], SE = 0.376, Table 5c, Fig. 2d). Species abundance was lower in areas with high precipitation variability (*β* = −0.307 [−0.524, −0.090], SE = 0.111, Table 5c, Fig. 2e) and in areas far away from water (*β* = −0.225 [−0.449, −0.001], SE = 0.114, Table 5c, Fig. 2f).

**Table 5:** Model predicting effects of environmental characteristics on the abundance of crop-exploiting bird species in the Indawgyi wetland ecosystem

Predictor variables	Estimate	SE	CI	
			Lower	Upper
a. Growing season				
**Intercept**	**3.582**	**0.232**	**3.127**	**4.038**
Habitat (forest)	−0.217	0.301	−0.801	0.378
Habitat (grassland)	−0.361	0.293	−0.936	0.214
Habitat (water)	−0.121	0.326	−0.761	0.517
Slope	−0.053	0.100	−0.247	0.141
Northness	−0.065	0.101	−0.262	0.132
Eastness	−0.099	0.087	−0.271	0.072
**Temperature seasonality**	−**0.237**	**0.092**	−**0.416**	−**0.057**
Precipitation seasonality	−0.174	0.093	−0.356	0.007
Distance to water	−0.033	0.100	−0.229	0.164
Distance to village	−0.089	0.097	−0.279	0.101
Random variable: Plot ID	SD = 0.363			
Random variable: sampling occasion	SD = 0.125			
b. Harvest season				
**Intercept**	**2.449**	**0.253**	**1.953**	**2.945**
Habitat (forest)	−0.343	0.345	−1.019	0.332
**Habitat (grassland)**	**0.691**	**0.339**	**0.026**	**1.357**
**Habitat (water)**	**0.493**	**0.129**	**0.240**	**0.746**
Slope	0.130	0.114	−0.093	0.353
Northness	−0.036	0.121	−0.272	0.202
Eastness	−0.130	0.101	−0.328	0.069
Temperature seasonality	−0.048	0.111	−0.266	0.169
Precipitation seasonality	0.003	0.106	−0.204	0.210
Distance to water	0.112	0.109	−0.101	0.326
Distance to village	0.090	0.105	−0.116	0.297
Random variable: Plot ID	SD = 0.599			
Random variable: sampling occasion	SD = 0.092			
c. After harvest season				
**Intercept**	**2.779**	**0.248**	**2.292**	**3.266**
Habitat (forest)	0.095	0.339	−0.569	0.761
Habitat (grassland)	−0.040	0.339	−0.703	0.624
**Habitat (water)**	**1.052**	**0.376**	**0.315**	**1.788**
Slope	−0.046	0.113	−0.268	0.176
Northness	0.172	0.126	−0.074	0.419
Eastness	0.093	0.118	−0.137	0.324
Temperature seasonality	0.018	0.119	−0.216	0.252
**Precipitation seasonality**	−**0.307**	**0.111**	−**0.524**	−**0.090**
**Distance to water**	−**0.225**	**0.114**	−**0.449**	−**0.001**
Distance to village	−0.030	0.106	−0.238	0.176
Random variable: Plot ID	SD = 0.035			
Random variable: sampling occasion	SD = 6.813e − 05			

Abundance in the avian community was used as a response variable whereas seven different environmental variables were used as predictors. Environmental variables are standardized with mean zero and a standard deviation. The analysis was based on a generalized linear mixed model fitted with negative binomial distribution using glmmTMB (Brooks et al. 2007). The identity of the bird sampling points, and sampling occasion were included as random factors. We present effect size of predictors, their standard errors and 95% confidence intervals, reference category for the habitat is Habitat (Agricultural land). CIs not overlapping zero in bold font

### Correlation Between the Spatio-Temporal Distribution of Crop-Exploiting Species and Other Species

We found that the richness of species that reduced agricultural yields was significantly correlated with the richness of other species which caused no yield losses across all seasons (richness: growing season: *r*_pearson_ = 0.318, *p* < 0.001, harvest season: *r*_pearson_ = 0.341, *p* < 0.001, after harvest season: *r*_pearson_ = 0.197, *p* = 0.031). But the correlation between abundance of crop-exploiting species and that of other species was significant only after harvest season (abundance: growing season: *r*_pearson_ = 0.043, *p* = 0.645, harvest season: *r*_pearson_ = 0.068, *p* = 0.462, after harvest season: *r*_pearson_ = 0.249, *p* = 0.006, Fig. S6).

### Spatio-Temporal Distribution of Six Species That Caused Serious Crop Losses

The evaluation metrics of our single species distribution models indicated that our models had good predictive ability. The AUC ranged from 0.700 to 0.942 and TSS ranged from 0.521 to 0.762 (Table 1).

**Fig. 3:**
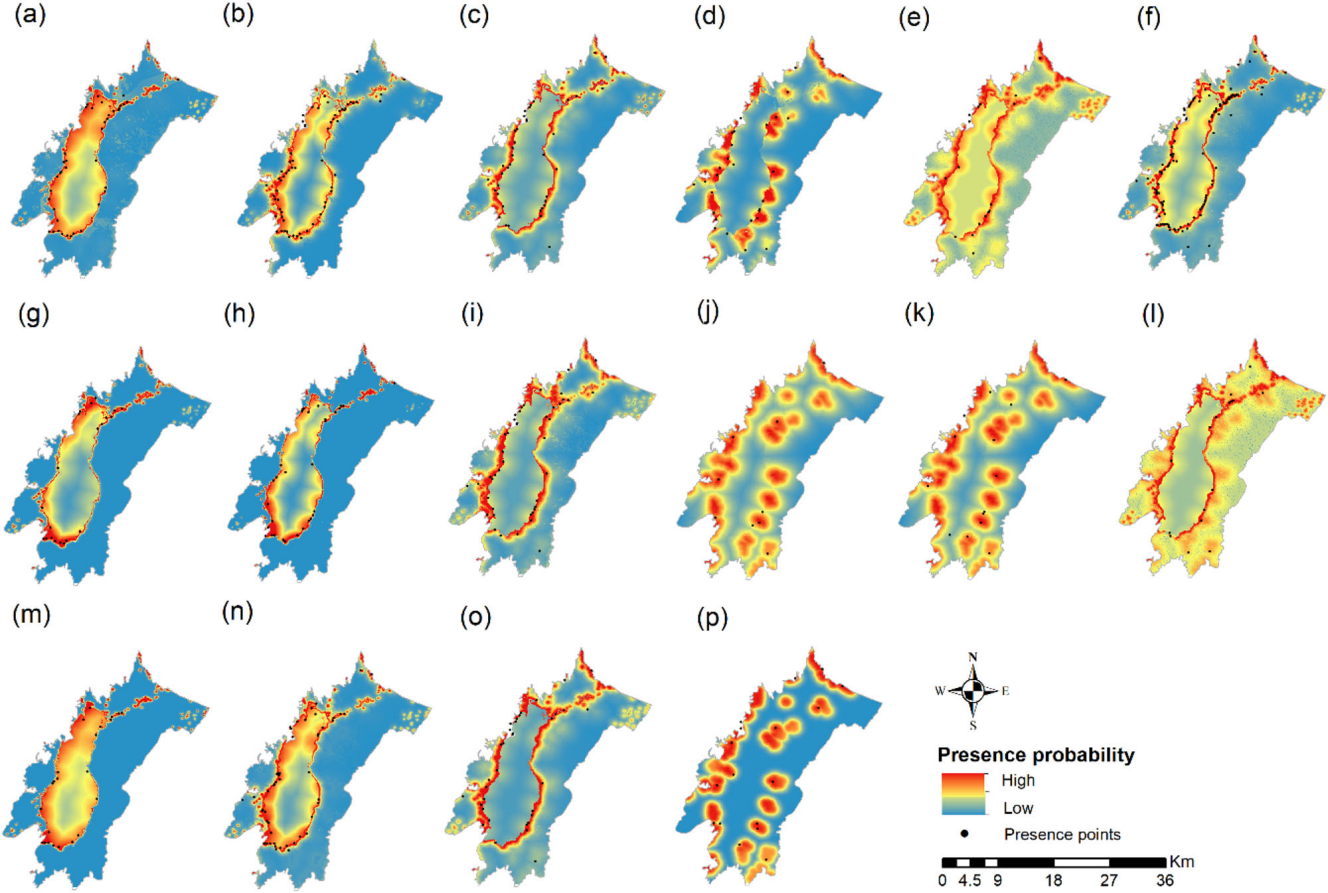
Spatial-temporal distribution of six species that caused the most damage to agricultural crops within the Indawgyi ecosystem. Figures are aligned season by rows and species by columns. Distribution during the growing season is in **a**–**f** (**a** Purple swamphen, **b** Lesser whistling duck, **c** Spotted dove, **d** Eurasian tree sparrow, **e** Scaly-breasted munia, **f** Baya weaver), whereas the distribution during harvest season is shown in **g**–**l** (**g** Purple swamphen, **h** Lesser whistling duck, **i** Spotted dove, **j** Eurasian tree sparrow, **k** Scaly-breasted munia, **l** Baya weaver). Distribution after the crop harvest season is described in **m**–**p** (**m** Purple swamphen, **n** Lesser whistling duck, **o** Spotted dove, **p** Eurasian tree sparrow). Distribution of Scaly-breasted munia and Baya weaver after the harvest season was excluded due to small sample size (<10 points). Presence probability increases from blue (low probability) to red (high probability). The black dots represent the species occurrence locations

**Fig. 4:**
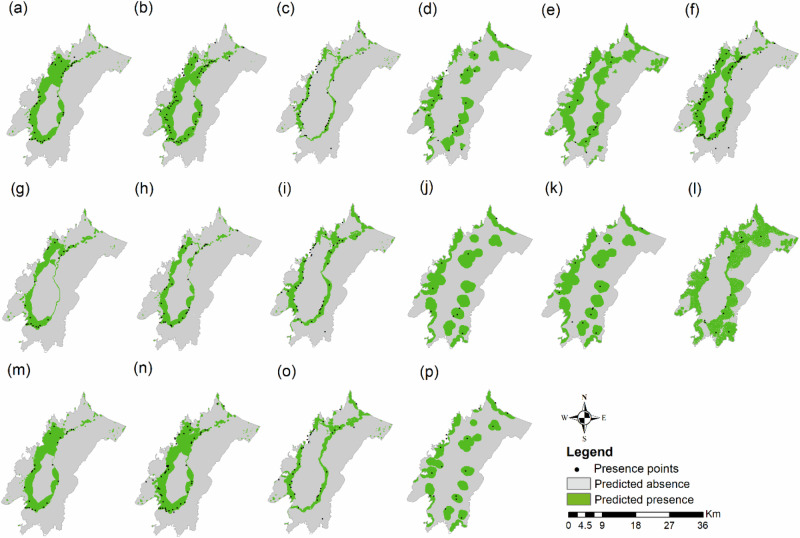
Presence areas of six species that caused the most damage to agricultural crops within the Indawgyi ecosystem. Figures are aligned season by rows and species by columns. Distribution during the growing season is in **a**–**f** (**a** Purple swamphen, **b** Lesser whistling duck, **c** Spotted dove, **d** Eurasian tree sparrow, **e** Scaly-breasted munia, **f** Baya weaver), whereas the distribution during harvest season is shown in **g**–**l** (**g** Purple swamphen, **h** Lesser whistling duck, **i** Spotted dove, **j** Eurasian tree sparrow, **k** Scaly-breasted munia, **l** Baya weaver). Distribution after the crop harvest season is described in **m**–**p** (**m** Purple swamphen, **n** Lesser whistling duck, **o** Spotted dove, **p** Eurasian tree sparrow). Distribution of Scaly-breasted munia and Baya weaver after the harvest season was excluded due to small sample size (<10 points). Presence areas are represented in green. The black dots represent the species occurrence locations

For the Purple swamphen (Figs. 3 and 4a, g, m), habitat types, temperature seasonality, distance to water, and distance to village were the main factors influencing the presence probability (Fig. S7). Distance to water was the most important variable in all seasons and contributed 65.6% in the growing season, 55.7% in the harvest and 86.5% after the harvest season (Table 3). The species presence probability was high within 500 m from sources of water and the effect was consistent in all seasons (Fig. S8). The effect of habitat types on the species occurrence was different among seasons, but the species presence probability was consistently the lowest in agricultural habitat in all seasons (Fig. S9). Species presence was also high in the proximity to the villages during the growing season (Fig. S10). Temperature seasonality had considerable effect in the harvest season and the presence probability was higher in areas with low to moderate temperature changes (Fig. S14).

For the Lesser whistling duck (Figs. 3 and 4b, h, n), habitat type, distance to water and distance to village were the main factors affecting its presence (Fig. S7). Distance to water was the most important variable in all seasons and contributed 65.7% in the growing season, 73.9% in the harvest and 61.6% after the harvest season (Table 3). The probability of presence was higher within 500 m from water and the effect was consistent across seasons (Fig. S8). However, the species response to habitat types varied seasonally and presence probability was the lowest in agricultural land in all seasons (Fig. S9). The effect of distance to village was consistent across seasons and species presence was higher near the villages.

For the Spotted dove (Figs. 3 and 4c, i, o), habitat type, northness, distance to water, and distance to village were the main factors affecting species presence (Fig. S7). Distance to water was the most important variable and contributed 40.3% in the growing season, 40.8% in the harvest season and 63.2% after the harvest season (Table 3). The species presence probability was higher within 1000 m from water and this effect was consistent across seasons (Fig. S8). The effect of habitat types varied seasonally (Fig. S9). Across all seasons, species presence was the highest in grassland, followed by forests and agriculture. The effect of distance to village was consistent across seasons and the presence probability was higher within 2500 m from the village (Fig. S10). The probability of species presence was lower in the north-facing aspect during the growing season (Fig. S12).

For the Eurasian tree sparrow (Figs. 3 and 4d, j, p), habitat type, distance to water, and distance to village were found to be the main factors influencing its presence (Fig. S7). Distance to village was the most important factor and contributed 73.4% in the growing season, 85.5% in the harvest season and 94.3% after the harvest season (Table 3). The probability of presence was higher near the villages, up to 1000 m (Fig. S10). The effect of distance to water was less important, but the species presence was higher near water (Fig. S8). Habitat effect was important only in the growing season and the species presence was highest in the grassland, followed by agricultural land and forest and the lowest in water (Fig. S9).

For the Scaly-breasted munia (Figs. 3 and 4e, k), habitat type, distance to water, and distance to village were the main factors influencing species presence (Fig. S7). The most important variable in the growing season was distance to water (contributed 49.7% to the model) and the most important variable in the harvest season was distance to village (contributed 72.3% to the model) (Table 3). Species presence probability was higher in areas close to water, especially during the growing season (Fig. S8). In both seasons, the presence probability was higher within 2000 m from the village. Habitat type is an important variable in the growing season and, but the presence probability was not different among habitats (Fig. S9).

For Baya weaver (Figs. 3 and 4f, l), habitat type, distance to water and distance to village were the main factors influencing species presence (Fig. S7). While distance to water was the most important variable in the growing season (contributed 71.2%), distance to village was the most important in the harvest season (contributed 53.1%) (Table 3). Both variables showed similar response patterns across seasons. Presence probability was higher close to water (within 500 m) and village (within 2500 m) (Figs. S8 and S10). Habitat type indicated seasonal patterns (Fig. S9). In the growing season, the species was the highest in grassland, followed by forests, water and agricultural land. In the harvest season, the presence was slightly higher grassland but was not very different among habitats.

### Effect of Crop Phenology and Environmental Conditions of the Farm on Diversity of Crop-Exploiting Species

During the growing season, species richness and abundance of crop-exploiting birds were higher in the seed broadcasting stage (richness: *β* = 0.466 [0.109, 0.823], SE = 0.182, Table 6a; abundance: *β* = 1.376 [0.556, 2.195], SE = 0.418, Table 7a). West-facing aspects (*β* = −0.323 [−0.644, −0.001], SE = 0.164, Table 7a) and low precipitation variability (*β* = −0.505 [−0.891, −0.118], SE = 0.197, Table 7a) favored for more abundances.

**Table 6:** Model predicting effects of crop stages and environmental factors in the agricultural habitat on the richness of crop-exploiting bird species in the Indawgyi wetland ecosystem

Predictor variables	Estimate	SE	CI	
			Lower	Upper
a. Growing season				
**Intercept**	**1.454**	**0.115**	**1.228**	**1.679**
**Stage (broadcasting)**	**0.466**	**0.182**	**0.109**	**0.823**
Stage (germinating)	0.064	0.190	−0.309	0.439
Stage (growing)	0.213	0.155	−0.090	0.517
Slope	−0.065	0.067	−0.196	0.067
Northness	0.021	0.065	−0.106	0.148
Eastness	0.081	0.068	−0.053	0.215
Temperature seasonality	−0.058	0.077	−0.209	0.093
Precipitation seasonality	−0.053	0.084	−0.219	0.113
Distance to water	−0.062	0.077	−0.213	0.090
Distance to village	−0.117	0.074	−0.264	0.029
Random variable: Plot ID	SD = 1.318e − 05			
Random variable: sampling occasion	SD = 1.818e − 06			
b. Harvest season				
**Intercept**	**1.471**	**0.213**	**1.052**	**1.890**
Stage (flowering)	−0.239	0.253	−0.736	0.258
Stage (ripening)	−0.266	0.238	−0.733	0.201
Stage (harvesting)	−0.569	0.326	−1.208	0.070
Slope	0.000	0.076	−0.149	0.150
Northness	−0.163	0.084	−0.327	0.001
Eastness	−0.013	0.087	−0.183	0.158
Temperature seasonality	0.122	0.097	−0.068	0.312
Precipitation seasonality	−0.189	0.103	−0.390	0.013
Distance to water	−0.008	0.089	−0.181	0.166
Distance to village	0.018	0.087	−0.152	0.188
Random variable: Plot ID	SD = 4.212e − 05			
Random variable: sampling occasion	SD = 5.022e − 08			
c. After harvest season				
**Intercept**	**0.832**	**0.197**	**0.446**	**1.218**
Stage (growing mustard)	−0.381	0.556	−1.471	0.709
Stage (growing soybean)	−0.044	0.271	−0.575	0.488
Stage (fired paddy)	−0.310	0.378	−1.051	0.430
Stage (fallow)	−0.937	0.618	−2.149	0.275
Slope	0.0279	0.071	−0.111	0.167
Northness	−0.178	0.097	−0.367	0.011
**Eastness**	**0.235**	**0.107**	**0.025**	**0.446**
Temperature seasonality	0.013	0.119	−0.221	0.247
Precipitation seasonality	−0.242	0.153	−0.542	0.058
**Distance to water**	−**0.356**	**0.138**	−**0.627**	−**0.084**
Distance to village	−0.021	0.135	−0.284	0.243
Random Variable: Plot ID	SD = 6.777e − 05			
Random Variable: sampling occasion	SD = 2.068e − 07			

Species richness in the avian community was used as a response variable whereas seven different environmental variables were used as predictors. Environmental variables are standardized with mean zero and a standard deviation. The analysis was based on a generalized linear mixed model fitted with Poisson distribution using glmmTMB (Brooks et al. 2007). The identity of the bird sampling points, and sampling occasion were included as random factors. We present effect size of predictors, their standard errors and 95% confidence intervals, reference category for the crop stage is Stage (Plowing) in the growing season, Stage (Growing) in the growing season, and Stage (Harvested) after the harvest season. CIs not overlapping zero in bold font

**Table 7:** Model predicting effects of crop stages and environmental factors in the agricultural habitat on the abundance of crop-exploiting bird species in the Indawgyi wetland ecosystem

Predictor variables	Estimate	SE	CI	
			Lower	Upper
a. Growing season				
**Intercept**	**3.131**	**0.241**	**2.659**	**3.604**
**Stage (broadcasting)**	**1.376**	**0.418**	**0.556**	**2.195**
Stage (germinating)	0.035	0.399	−0.747	0.816
Stage (growing)	0.028	0.333	−0.626	0.681
Slope	0.006	0.163	−0.313	0.325
Northness	−0.047	0.160	−0.360	0.266
**Eastness**	−**0.323**	**0.164**	−**0.644**	−**0.001**
Temperature seasonality	0.001	0.182	−0.356	0.358
**Precipitation seasonality**	−**0.505**	**0.197**	−**0.891**	−**0.118**
Distance to water	−0.183	0.185	−0.546	0.180
Distance to village	−0.116	0.168	−0.444	0.212
Random variable: Plot ID	SD = 5.177e − 01			
Random variable: sampling occasion	SD = 2.885e − 05			
b. Harvest season				
**Intercept**	**3.009**	**0.481**	**2.066**	**3.953**
Stage (flowering)	−0.790	0.517	−1.804	0.224
Stage (ripening)	−0.518	0.485	−1.470	0.433
Stage (harvesting)	−0.929	0.619	−2.143	0.286
Slope	0.174	0.163	−0.146	0.492
Northness	−0.280	0.167	−0.606	0.047
Eastness	−0.155	0.182	−0.512	0.203
Temperature seasonality	0.113	0.196	−0.271	0.497
**Precipitation seasonality**	−**0.468**	**0.218**	−**0.894**	−**0.041**
Distance to water	−0.003	0.192	−0.379	0.372
Distance to village	0.138	0.173	−0.202	0.478
Random variable: Plot ID	SD = 5.622e − 01			
Random variable: sampling occasion	SD = 4.469e − 05			
c. After harvest season				
**Intercept**	**1.909**	**0.588**	**0.756**	**3.062**
**Stage (growing mustard)**	−**1.302**	**0.631**	−**2.541**	−**0.063**
Stage (growing soybean)	0.157	0.385	−0.598	0.912
**Stage (fired paddy)**	−**0.800**	**0.204**	−**1.199**	−**0.401**
Stage (fallow)	−1.378	1.475	−4.268	1.511
Slope	−0.036	0.219	−0.465	0.393
Northness	−0.299	0.279	−0.846	0.248
**Eastness**	**0.625**	**0.298**	**0.040**	**1.209**
Temperature seasonality	−0.131	0.324	−0.766	0.504
Precipitation seasonality	−0.359	0.384	−1.112	0.394
Distance to water	−0.578	0.328	−1.221	0.064
Distance to village	0.127	0.325	−0.510	0.764
Random variable: Plot ID	SD = 1.2714			
Random variable: sampling occasion	SD = 0.2573			

Species abundance in the avian community was used as a response variable whereas seven different environmental variables were used as predictors. Environmental variables are standardized with mean zero and a standard deviation. The analysis was based on a generalized linear mixed model fitted with negative binomial distribution using glmmTMB (Brooks et al. 2007). The identity of the bird sampling points, and sampling occasion were included as random factors. We present effect size of predictors, their standard errors and 95% confidence intervals, reference category for the crop stage is Stage (Plowing) in the growing season, Stage (Growing) in the growing season, and Stage (Harvested) after the harvest season. CIs not overlapping zero in bold font

During the harvest season, crop phenology had no effect on species richness (Table 6b). However, species abundance decreased with increasing precipitation seasonality (*β* = −0.468 [−0.894, −0.041], SE = 0.218, Table 7b).

After the harvest season, species richness was higher in farms close to water (*β* = −0.356 [−0.627, −0.084], SE = 0.138, Table 6c). However, species abundance was lower in mustard crops (*β* = −1.302 [−2.541, −0.063], SE = 0.631, Table 7c), fired paddy fields (*β* = −0.800 [−1.199, −0.401], SE = 0.204, Table 7c). East-facing aspect had positive effects on both richness and abundance (richness: *β* = 0.235 [0.025, 0.446], SE = 0.107, Table 6c, abundance: *β* = 0.625 [0.040, 1.209], SE = 0.298, Table 7c).

## Discussion

Our study demonstrates that the distribution of crop-exploiting avian species varied spatially and temporally in response to seasonal environmental changes of the wetland ecosystem. Analyses of species richness and abundance as well as species-specific distribution models indicated that habitat type is the common important variable influencing the distribution of crop-exploiting birds. However, the influences of topographic, climatic, and anthropogenic variables were different between overall pattern and species-specific distribution. These findings raised the discussion about how the ecological response of crop-exploiting avian species varied seasonally with landscape and bioclimatic changes, how these responses are different between the overall pattern and species-specific patterns, and how to allocate management efforts to promote coexistence between bird conservation and agricultural production.

### Spatio-Temporal Responses of Crop-Exploiting Avian Species to Environmental Factors

#### Growing Season

During the growing season, the species richness was higher in agricultural land, grassland, and water but was the lowest in forest. As the crop growing season overlaps with the breeding season, the observed pattern could be attributed to resource required for breeding success. Vegetation cover that provides shelter for safe nesting, invertebrates that are rich in protein content and humid environment that enhances incubation are fundamental requirements for breeding birds (Zufiaurre et al. 2017). Grassland and aquatic vegetation in water seem to be ideal habitats for most bird species to fulfill these requirements (Ma et al. 2010; Maphisa et al. 2019). Agriculture lands are also flooded during this season, and the abundance of rice grains and aquatic invertebrates could be attractive to bird as foraging habitats (Parejo et al. 2019). Results also indicated that crop-exploiting species were more abundant in the seed broadcasting stage (Tables 6 and 7). Previous studies also found that flooding regimes influence prey densities, which in turn causing fluctuations in the densities of birds foraging in rice fields (Amano et al. 2008; Ibáñez et al. 2010). Although forests were used by fewer species, the abundance was not different from the other habitats, suggesting that forest habitats were also used by higher number of forest-dwelling species such as doves and parakeets (Zufiaurre et al. 2017). However, both species richness and abundance decreased with seasonal increase in temperature variation (Figs. 1c and 2c, d). As variation in temperature directly influences the chick survival, the species seems to prefer stable climatic conditions (Girma et al. 2017; Monge et al. 2023; Şekercioğlu et al. 2012). The effects of topo-climatic variables were also significant in the species that occurred only in the agricultural habitat (Tables 5 and 6). We found that species abundance was higher in areas with low precipitation variation and in west-facing aspects, most likely because it receives more solar radiation and had an indirect effect on primary productivity, plant growth, and invertebrate biomass (Davies et al. 2007; Ding et al. 2006).

When investigating the distribution of the six species that caused severe damage to agricultural crops, distance to water was found as the most important factor for all species except Eurasian tree sparrow. Irrespective of whether the species is wetland specialist or generalist, they displayed a higher presence probability in the proximity to water (Table 3 and Fig. S8). Grassland and riparian forests are found as commonly used habitats by all species, although the importance of habitat was different among species (Fig. S9). Grassland and riparian forests are adjacent to water and are rich in invertebrate foods that are essential to laying females and growing nestlings. Therefore, nesting in these habitats has advantages like lower energetic costs for movement between breeding and foraging sites and predation pressures during chick rearing (Canaveli et al. 2014). Among the species, Spotted dove, Eurasian tree sparrow, and Scaly-breasted munia showed higher preference to agricultural land than Purple swamphen, Lesser whistling duck and Baya weaver (Fig. S9). Although the effect of distance to village was not very strong as those of distance to water and habitat, all species revealed their presence near the villages (Fig. S10). In Eurasian tree sparrow, distance to village was the main factor influencing its presence. This indicates that all these species are adaptable to human disturbances when the natural or semi-natural habitats are available in their surroundings (Li et al. 2019; Rahlin et al. 2022).

#### Harvesting Season

During the crop harvesting season, overall species richness and abundance were higher in grassland and water (Figs. 1 and 2). During this season, grasslands and riparian forests are inundated due to seasonal changes in hydrological regime and the expansion of the waterbody (Forest Department 2018; Tozer et al. 2010). Additionally, wild rice in grassland were ripen, making this habitat rich in resources that can be exploited by a variety of avian functional groups (Htay et al. 2023b). Furthermore, migratory species that had higher affinity to wetland arrived and aggregated in the grassland and water (for instances, crane, shelduck and goose) making the richness and abundance higher in these habitats during this season (McPherson and Jetz 2007). Although the forests are also flooded and used by some waterbirds like Lesser whistling duck, the resources in this habitat are not as diverse as in natural wetland habitats and most species seem to prefer grassland and water habitat. Fewer number of forest-dwelling species in our bird community may be one reason for this. Although the agricultural land had abundant resources due to rice ripening, these resources are homogenous (Htay et al. 2023b). Furthermore, the crop fields were drained and plant and animal biomass that most species preferred were not as diverse as in the growing season. So, it might be mostly used by granivorous species (like passerines) and some generalists (Natuhara 2013). Habitat use of the six species that impacted most to the crop production also indicated that the presence probability of Spotted dove, Eurasian tree sparrow, Scaly-breasted munia and Baya weaver were much higher in agricultural land (Fig. S9). For these species, the effect of distance to village and agricultural use increased. The other two species; Purple swamphen and Lesser whistling duck, however, showed the opposite pattern, where the distance to water was the most important variable (Fig. S7) and indicated higher presence probability in grassland and water (Fig. S9). Although these species were still found near the village, the effect of distance to village was less important in this season than the previous season (Table 3). Hunting pressures over large waterbirds during the crop harvesting seasons, which overlaps with migration season could also be one possible factor for weak effect of distance to villages on their distribution (Htay et al. 2023a).

#### After Harvest Season

After the crop harvest season, both richness and abundance were high only in the water habitat. As this season overlaps with summer, availability of water seems to be a major limiting factor in the distribution of biological communities and for agricultural activities (Weller 1999; Zhang et al. 2023). In this season, rice fields are already harvested, and the new crops are not grown in most part of the region due to lack of rains. Only some fields near the alluvial areas in the northeastern part grow second crop either soybean or mustard. So, most harvested rice fields are fallowed, or fired or prepared for the next growing season (Htay et al. 2022). However, agricultural activities had no positive effects on the richness and abundance of crop-exploiting birds. Results indicated species abundances were lower in mustard and fired paddy fields (Tables 6 and 7). Water desiccation, higher rates of evapotranspiration, changes in inundation patterns and soil moistures also altered vegetation structure of the grassland to be tall grass-dominated and those of the forest to be shrub dominated (Weller 1999). Previous studies found that bird feed less in taller and dense vegetation structure because of limited prey capture rates (Ibáñez et al. 2010). Therefore, the resources that the species prefer might be abundantly available near water and both richness and abundance seem to be higher in this habitat. Analysis of the species from the agricultural habitat also indicated that a higher abundance near water and in the east-facing aspects with low sunlight and more moisture (Tables 5 and 6). Furthermore, the species abundance was lower in areas with precipitation seasonality (Fig. 2h, i). This finding indicates that, even outside the breeding season, the species seems to be physiologically sensitive to climatic variations (Li et al. 2019) and habitats that are easily accessible to replace evaporative water loss and that can provide microclimatic buffering seems to be important (Monge et al. 2023; Şekercioğlu et al. 2012). Distribution of species that caused most crop losses also indicated that distance to water was the most important factor determining the species presence (in Purple swamphen, Lesser whistling duck, and Spotted dove) (Fig. S7). These species used natural habitats more than agricultural habitats (Fig. S9). However, for Eurasian tree sparrow, distance to water and habitat type were not as important as distance to village. Therefore, this species seems to exploit a wide range of supplementary food and water sources in the villages (Zufiaurre et al. 2016).

### Differences in Environmental Effects Between Overall Patterns and Species-Specific Patterns

Our results indicated that the importance of habitat factor is consistent between overall pattern and single species models. However, the effects of topographic, anthropogenic and climatic variables were not consistent between overall model and species-specific models. We found that distance to village was an important variable in the single species distribution modeling, but it was not significant in predicting overall richness and abundance (Tables 3, 4 and 5). As our communities included a mix of wetland specialist and generalists that had higher adaptation to wetland edges and terrestrial habitats, this might be related to variation in ecological preferences among individual species and some of these species-specific effects might be masked when we analyzed the patterns using overall richness and abundance (McPherson and Jetz 2007). However, all six species that caused serious crop damage revealed higher presence probabilities near the villages, although the contribution of this variable varied among species (Fig. S10). These species (in particular Spotted dove, Eurasian tree sparrow, Scaly-breasted munia, and Baya weaver) possessed wider habitat and dietary breadth (Canavelli et al. 2014; Studholme et al. 2018) and thus were more tolerant to anthropogenic influences (Briceño et al. 2019). Previous studies have also found that birds’ tolerance to human influences is higher among habitat generalists and smaller species that flock in large groups. These species possess wider ecological niches, quicker predator detection and better flight efficiency (Møller and Díaz 2018; Neate-Clegg et al. 2023). Our results indicated that the climatic variables had weak influences on the species that caused serious yield losses although they had strong significant effects on the overall richness and abundance (Tables 3, 4 and 5). In line with our findings, previous global meta-analyses about human–bird interactions reported that species adapted to novel environments and climatic variation are more involved in human–bird conflict (Araneda et al. 2022; Fox et al. 2017). Therefore, with increasing global climate change, farmers are more likely to be economically susceptible from serious crop-exploiting species coupled with uncertainty in agricultural productions and the best management solutions play a key role to promote coexistence (Araneda et al. 2022).

### Management Implications for Avifauna Conservation

We found that the overall richness of crop-exploiting species significantly correlated with that of other species in all seasons (Fig. S6). Although the strength of correlation was lower, this finding indicates that the species from the two groups co-occur in our sampling points and suggest that the failure to reduce the impact of crop-exploiting species on the farmers’ agricultural production and their unsustainable control method will probably impact other non-targeted groups (Angkaew et al. 2022). Sustainable management measures are important not only from the conflict resolution perspectives, but also from conservation perspectives because some of the crop-exploiting species are experiencing population decline globally and illegal killing locally (Htay et al. 2023b). If management interventions are to be implemented, areas close to water (especially within 1 km) should be the priority areas to reduce the impact of the species that caused most damages. Northern and western parts of the wetland are more important because four out of the six serious crop-exploiting species were high (Purple swamphen, Lesser whistling duck, Scaly-breasted munia, Baya weaver) in these areas (Figs. 3 and 4).

During the crop growing season, management should focus on the seed broadcasting stage because they attract a lot of crop-exploiting species due to flooding and the high resource abundance. During the crop harvest season, although farmers complain about more bird damages (Htay et al. 2022), we found that richness and abundance of the species were higher in grassland and water than agricultural habitat (Figs. 1d and 2e). Among the bird species causing more damage, the occurrence of Purple swamphen and Lesser whistling duck were higher in natural wetland habitats than agricultural habitat. Although Spotted dove, Eurasian tree sparrow, Scaly-breasted munia and Baya weaver were found high in agricultural land, they are also widespread in other habitats (Fig. S9). Therefore, in the crop harvesting season, conservation of natural wetland habitats and improved habitat conditions would act as “refuge field” and probably reduce species movement to the farms and conflicts with farmers. However, we are cautious about this conclusion because of the inconsistency between our findings and farmers reporting. This could be explained by the difference in species detection between our species surveys and farmers’ observations. The discrepancy between the two sources is more obvious in waterbirds (Htay et al. 2022). Farmers reported waterbird damage was higher during the late evening and at night and their reporting was in line with waterbird habitat uses in rice fields of Asia and elsewhere (Parejo et al. 2019). But our species survey that was conducted during the daytime was by default unable to monitor nocturnal behavior. Thus, agricultural areas close to grassland and water habitat might potentially experience conflict due to the spillover effect. Therefore, in such areas, conservation management should find alternatives for coexistence such as economic compensation for crop loss. Future research using satellite tracking and multiscale analyses (i.e., that account for influence of habitat factors at different spatial scales) is recommended to examine this further (Nilsson et al. 2018; Parejo et al. 2019). A follow-up study about the compensatory growth of rice plants to bird herbivory (for instance anti-herbivory mechanisms that can regrow new shoots, flower and seed after being foraged by birds) may contribute to understand why farmers are more sensitive to crop loss during the harvest season than in the growing season (Clausen et al. 2022; Marco-Méndez et al. 2015). After the harvest season, intervention is not as important as in the previous two growth seasons because most of the crop fields were fallowed. And among the fields that grow crops, there were negative effects of later crops on species richness and abundance. However, follow-up studies on the behavioral and ecological response of crop-exploiting bird species to conservation management practices (e.g., habitat management, deterrents) would provide further insights into their habitat use (Enos et al. 2021). Additionally, the inclusion of vegetation productivity metrics such as normalized difference vegetation index (NDVI) into species distribution modeling would enhance our predictive capabilities (Calamari et al. 2018). Unfortunately, we could not include NDVI in this study due to the limited availability of seasonal data from this area.

Our findings on the spatio-temporal distribution of crop-exploiting bird species have significant implications for conservation planners. These insights can guide the allocation of effective management and coexistence strategies to achieve sustainable socio-ecological outcomes in internationally important wetland areas. While this study utilizes a case study approach, the findings are generalizable to other wetlands in Myanmar with similar socio-ecological contexts. Furthermore, our findings can be associated to other studies in rice-dominated wetlands across Southeast Asia to enhance regional bird conservation initiatives (Angkaew et al. 2022).

## Supplementary information

Supplementary Information
