# Supplementary material for: Environmental Factors Affecting Spatio-Temporal Distribution of Crop-Exploiting Species: Implications for Coexistence Between Agricultural Production and Avifauna Conservation in Wetlands
Source: Environ Manage. 2024 Aug 4;74(4):664–83. doi: 10.1007/s00267-024-02028-7 (PMC11393005; doi:10.1007/s00267-024-02028-7)
Supplement: Supplementary file 1 — Supplementary Information [file 267_2024_2028_MOESM1_ESM.docx]

**Supplementary Materials**

**Environmental factors affecting spatio-temporal distribution of crop-exploiting species: Implications for coexistence between agricultural production and avifauna conservation in wetlands**

Thazin Htay ^a,b*,1^, Kyaw Kyaw Htoo ^c,2^ , Eivin Røskaft ^a, 3^, Thor Harald Ringsby ^a,d,§,4^, Peter Sjolte Ranke ^a,d,e,§,5^

^a^ Department of Biology, Norwegian University of Science and Technology (NTNU), Realfagbygget, NO-7491, Trondheim, Norway

^b^ Nature and Wildlife Conservation Division, Forest Department, Ministry of Natural Resources and Environmental Conservation, Nay Pyi Taw, Myanmar

^c^ Division of Forest and Biomaterials Science, Graduate School of Agriculture, Kyoto University, Japan

^d^ Centre for Biodiversity Dynamics (CBD), Department of Biology, Norwegian University of Science and Technology (NTNU), NO-7491 Trondheim, Norway

e BirdLife Norway, NO-7012 Trondheim, Norway

§ Joint senior authors

^1^ ORCID: 0000-0002-9995-7712

^2^ ORCID: 0000-0003-3144-0028

^3^ ORCID: 0000-0003-0262-8443

^4^ ORCID: 0000-0001-9089-7592

^5^ ORCID: 0000-0003-3757-8626

Land cover classification

In this study, we employed the Google Earth Engine (GEE) for the land cover classification (<https://earthengine.google.com/>). GEE is an open-source cloud-based platform that allows users to access and analyze huge amounts of satellite data archived in the google server. Because of user-friendliness, large-scale computing, and well-developed algorithms for many kinds of geospatial analysis, it is becoming a popular tool in recent years (Gorelick et al. 2017).

The general workflow for land cover classification is mainly composed of four steps (Fig. S1). Firstly, the Landsat 9 satellite images (30x30 m resolution) were chosen from the GEE archived datasets for its improved sensor, data quality and updated information than for previous ancestors. We limit the image collection by creating a bounding box that encompass the whole landscape of our study area. Although images were available from February 2022, when the image collections were filtered with cloud cover less than 10 % for the study area, a period between November 2022, and April 2023, dates met the criteria. Therefore, we chose the available images (29) between those periods. We selected the Tier 1 Level-2 product, which had undergone preprocessing procedures including atmospheric correction and computation of surface reflectance. Then, specific scaling factors provided for the Landsat 9 bands were applied to ensure the data quality and mitigate the artifacts. Subsequently, the calculation of the median value for each pixel across the entire set of images was performed. The resulting image represents the pixel-wise median value of all images in the collection. Calculating the median is a widely adopted approach to obtain a representative image from an image collection as it helps reduce the impact of outliers and provides a more stable and comparable estimation of the overall pixel values across the images collection dates (Tassi. et al. 2020; Phan et al. 2020).

Next, we prepared the training datasets for random forest supervised land cover classification. Random forest is considered as one of the most widely used and powerful algorithms in land cover analysis (Millard & Richardson 2015; Amani et al. 2019; Phan et al. 2020). It applies a pixel-based discrimination method between land cover classes by learning the training data provided by the user. Therefore, training features for distinct land cover classes namely Forest (476), Crop (527), Grassland (180) and Water (120) were created. To ensure each data class, Red Green Blue (RGB) image and other composite layers specific for different land cover classes and the high-resolution Google Maps were displayed and data were visually identified and labelled by using GEE interface. In total 1303 features were able to construct as a comprehensive training dataset. This approach is commonly applied in literature (Hansen et al. 2008; Tassi et al. 2020). The way we choose our training data has a big impact on how accurate our classification results are (Millard & Richardson, 2015). So, we designed our training data selection process in a way that makes sure the proportions of different classes in the training data match the actual proportions of those classes in the real landscape. This helps us create training data that better represents the real-world conditions, leading to more reliable and accurate classification results. Created training data were then randomly separated into two sets: one for training land cover classification model (70 % of data) and the other for testing its accuracy (30 % of data).

We then used Randon Forest classifier algorithm available in GEE using the training data. The classifier learns to associate the input band information with the corresponding land cover classes from the training dataset and classified into land cover categories as it has learned.

An accuracy assessment between the classified map and validation data was performed, and we achieved the overall accuracy of 0.9653. The details of accuracy assessment for each class are provided in Table S1. The constituted landcover classes were agriculture (55.3%), forest and woody scrub (8.3%), grassland (10%) and water (26.4%).

The digital elevation model (DEM) of 30 m resolution for the study area was downloaded from the NASA Shuttle Radar Topography Mission (SRTM) (Farr et al. 2007). From the DEM, we calculated the Aspect and Slope Models using “Spatial Analyst” tool in ArcMap v.10.8.2.

References

Amani, M., Mahdavi, S., Afshar, M., Brisco, B., Huang, W., Mohammad Javad Mirzadeh, S., ... & Hopkinson, C. (2019). Canadian wetland inventory using Google Earth Engine: The first map and preliminary results. Remote Sensing, 11(7), 842.

de Sousa, C., Fatoyinbo, L., Neigh, C., Boucka, F., Angoue, V., & Larsen, T. (2020). Cloud-computing and machine learning in support of country-level land cover and ecosystem extent mapping in Liberia and Gabon. PLoS One, 15(1), e0227438.

Farr, T. G., et al. (2007), The Shuttle Radar Topography Mission,Rev. Geophys.,45, RG2004.

Gorelick, N., Hancher, M., Dixon, M., Ilyushchenko, S., Thau, D., & Moore, R. (2017). Google Earth Engine: Planetary-scale geospatial analysis for everyone. Remote sensing of Environment, 202, 18-27.

Hansen, M. C., Roy, D. P., Lindquist, E., Adusei, B., Justice, C. O., & Altstatt, A. (2008). A method for integrating MODIS and Landsat data for systematic monitoring of forest cover and change in the Congo Basin. Remote Sensing of Environment, 112(5), 2495-2513.

Millard, K., & Richardson, M. (2015). On the importance of training data sample selection in random forest image classification: A case study in peatland ecosystem mapping. Remote sensing, 7(7), 8489-8515.

Phan, T. N., Kuch, V., & Lehnert, L. W. (2020). Land cover classification using Google Earth Engine and random forest classifier—The role of image composition. Remote Sensing, 12(15), 2411.

Tassi, A., & Vizzari, M. (2020). Object-oriented lulc classification in google earth engine combining snic, glcm, and machine learning algorithms. Remote Sensing, 12(22), 3776.


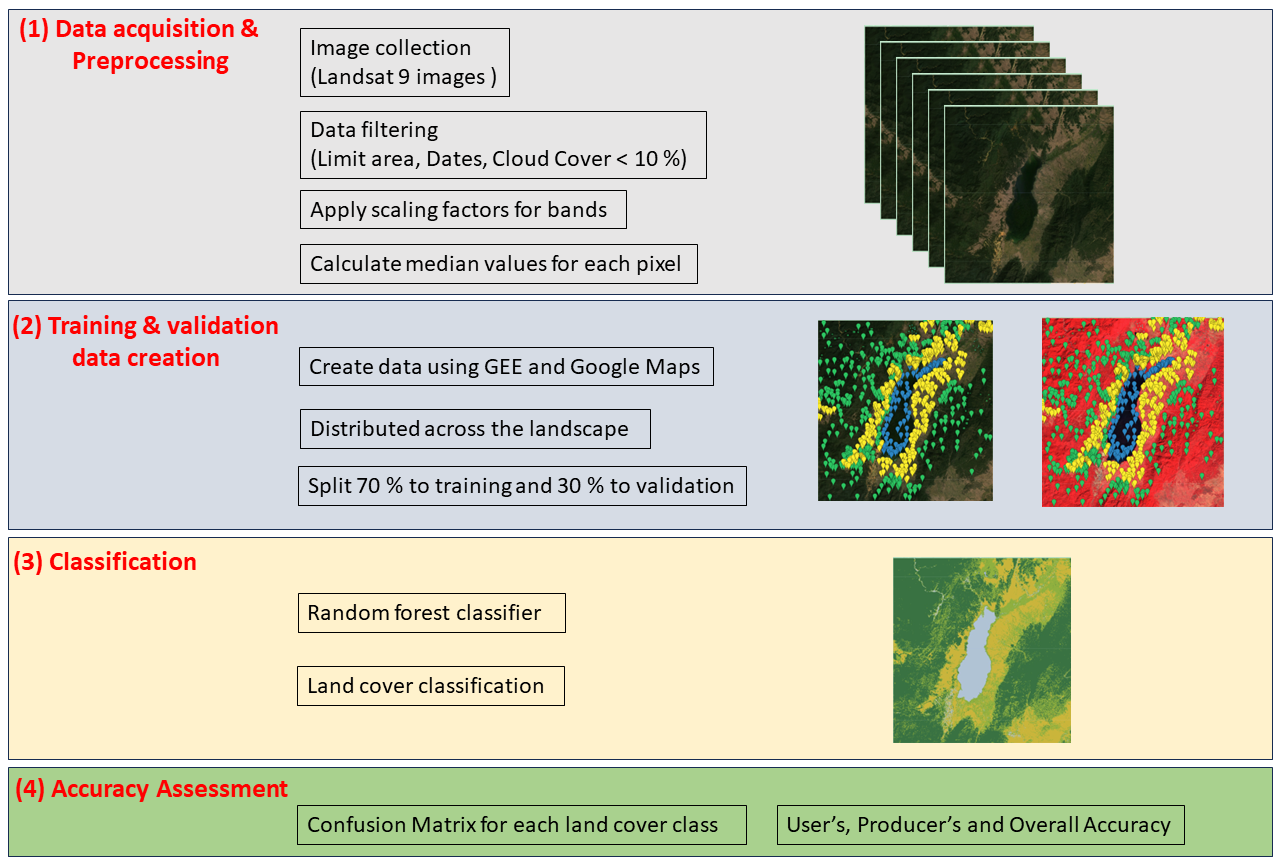


Fig. S1. General workflow of land cover analysis in Google Earth Engine (GEE)

Table S1. Accuracy assessment of the land cover classification results

|  | Land cover classes | Validation data | | | |  | |
| --- | --- | --- | --- | --- | --- | --- | --- |
|  |  | Water | Forest | Agriculture | Grassland | Row total | User's accuracy (%) |
| Classification data | Water | 51 | 1 | 0 | 13 | 65 | 78.46 |
|  | Forest | 1 | 1924 | 2 | 30 | 1957 | 98.31 |
|  | Agriculture | 2 | 3 | 133 | 24 | 162 | 82.10 |
|  | Grassland | 3 | 23 | 7 | 929 | 962 | 96.57 |
|  | Column total | 57 | 1951 | 142 | 996 | 3146 |  |
|  | Producer's accuracy (%) | 89.47 | 98.62 | 93.66 | 93.27 |  | Overall accuracy = 96.53 |


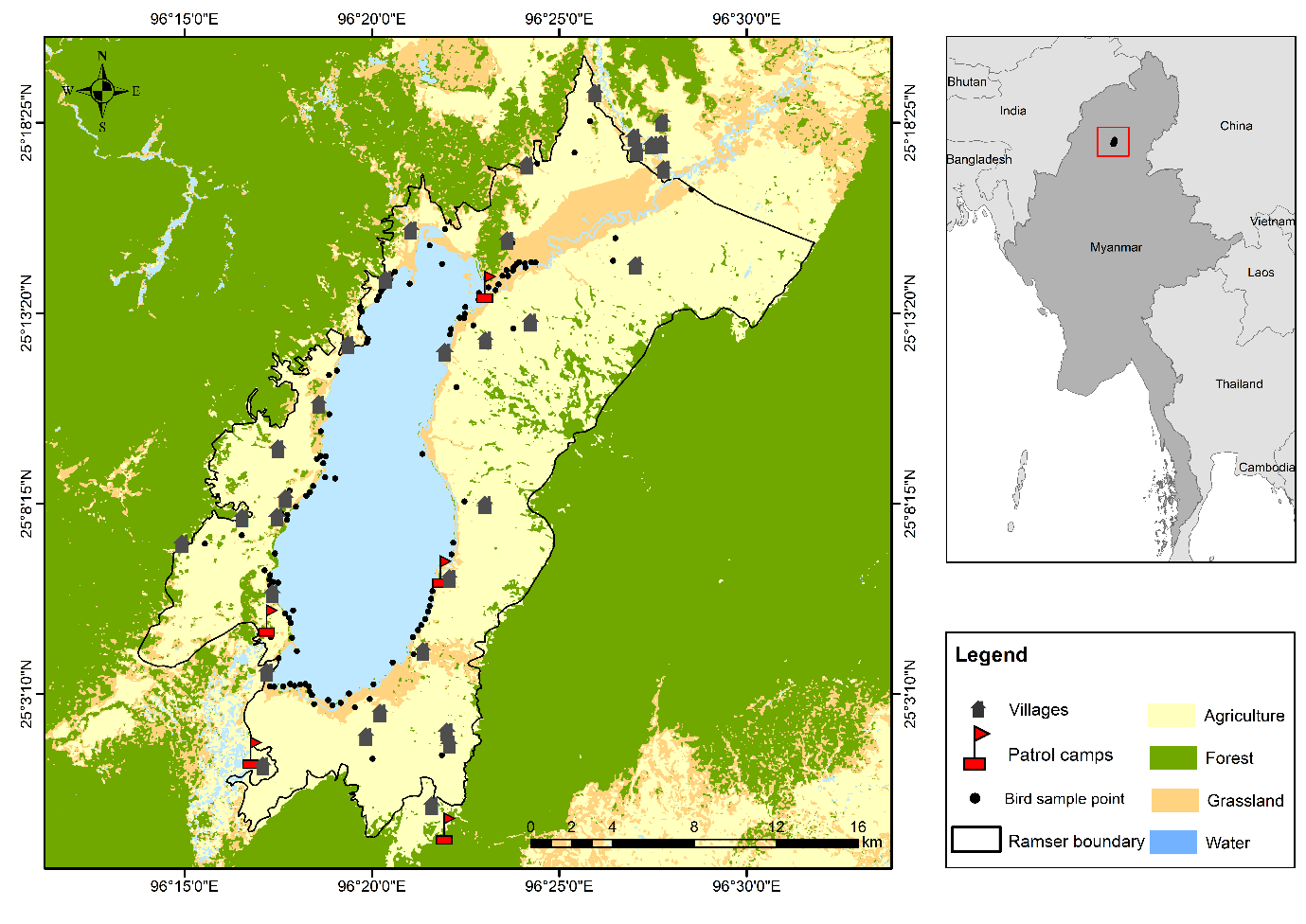


Fig. S2. Location of the Indawgyi wetland ecosystem in Myanmar (red square in the upper inset) along with distribution of major land cover types, study villages and bird sampling points.


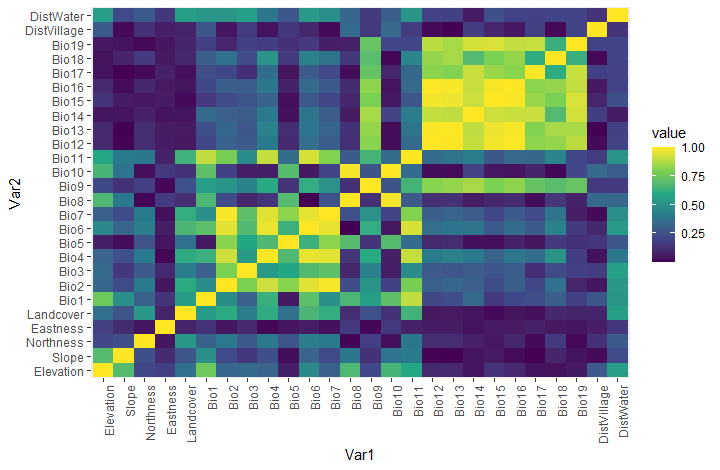


Fig. S3. Correlation tests among candidate environmental predictors used to model distribution of crop-exploiting avian species. BIO1 = Annual Mean Temperature, BIO2 = Mean Diurnal Range, BIO3 = Isothermality, BIO4 = Temperature Seasonality, BIO5 = Max Temperature of Warmest Month, BIO6 = Min Temperature of Coldest Month, BIO7 = Temperature Annual Range, BIO8 = Mean Temperature of Wettest Quarter, BIO9 = Mean Temperature of Driest Quarter, BIO10 = Mean Temperature of Warmest Quarter, BIO11 = Mean Temperature of Coldest Quarter, BIO12 = Annual Precipitation, BIO13 = Precipitation of Wettest Month, BIO14 = Precipitation of Driest Month, BIO15 = Precipitation Seasonality, BIO16 = Precipitation of Wettest Quarter, BIO17 = Precipitation of Driest Quarter, BIO18 = Precipitation of Warmest Quarter, BIO19 = Precipitation of Coldest Quarter, DistVillage = Distance to nearest village, DistWater = Distance to nearest source of water


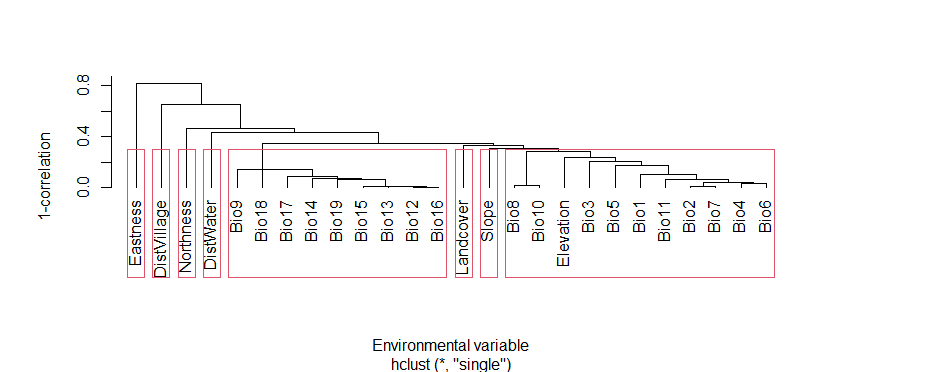


Fig. S4. Clustered dendrogram among environmental variables that are aggregated according to their correlation coefficient. The correlation threshold is r _person_ > 0.7


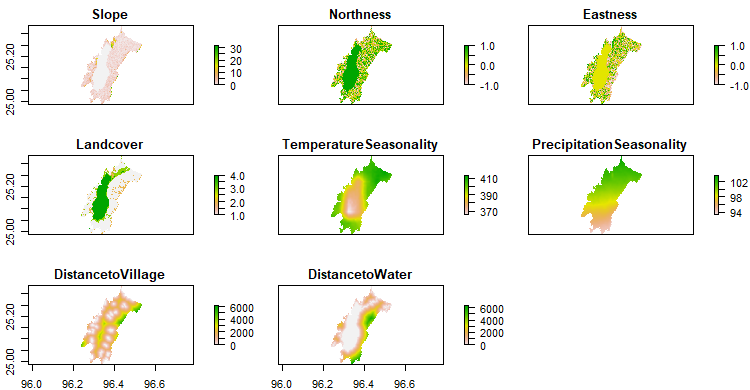


Fig. S5. Environmental variables used in the species distribution modelling of crop-exploiting avian species


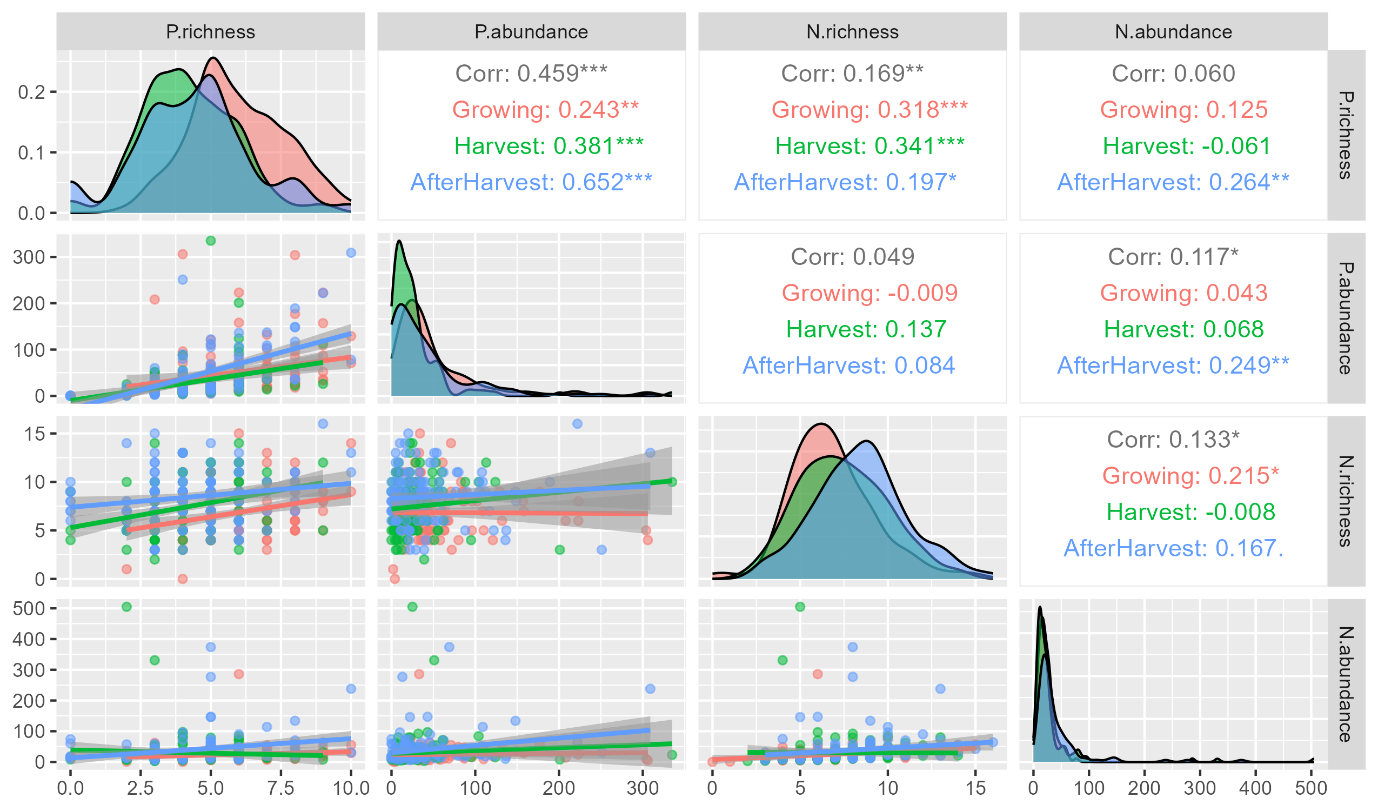


Fig. S6. Correlation test between the richness and abundance of crop-utilizing species with those of non-crop utilizing species for each season


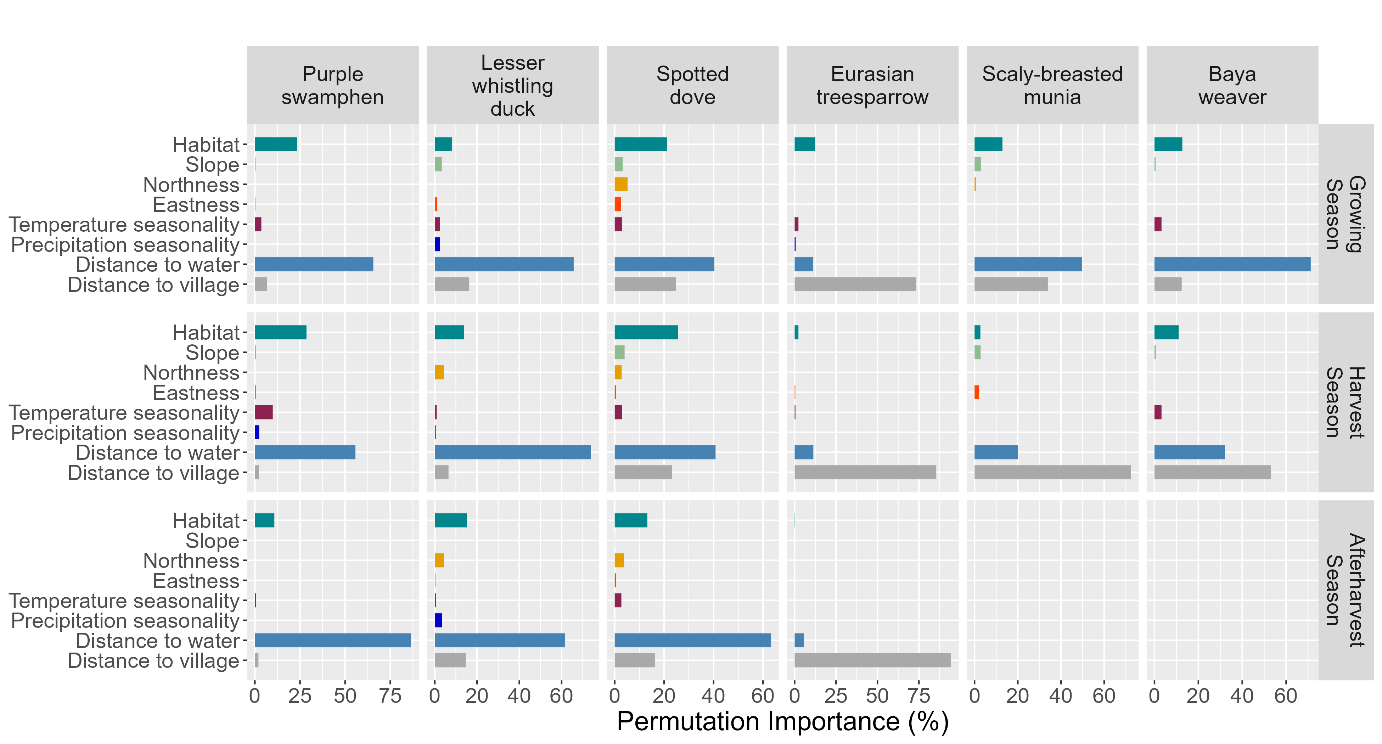


Fig. S7. Permutational importance of environmental variables in the distribution model of the six bird species that caused serious crop damage


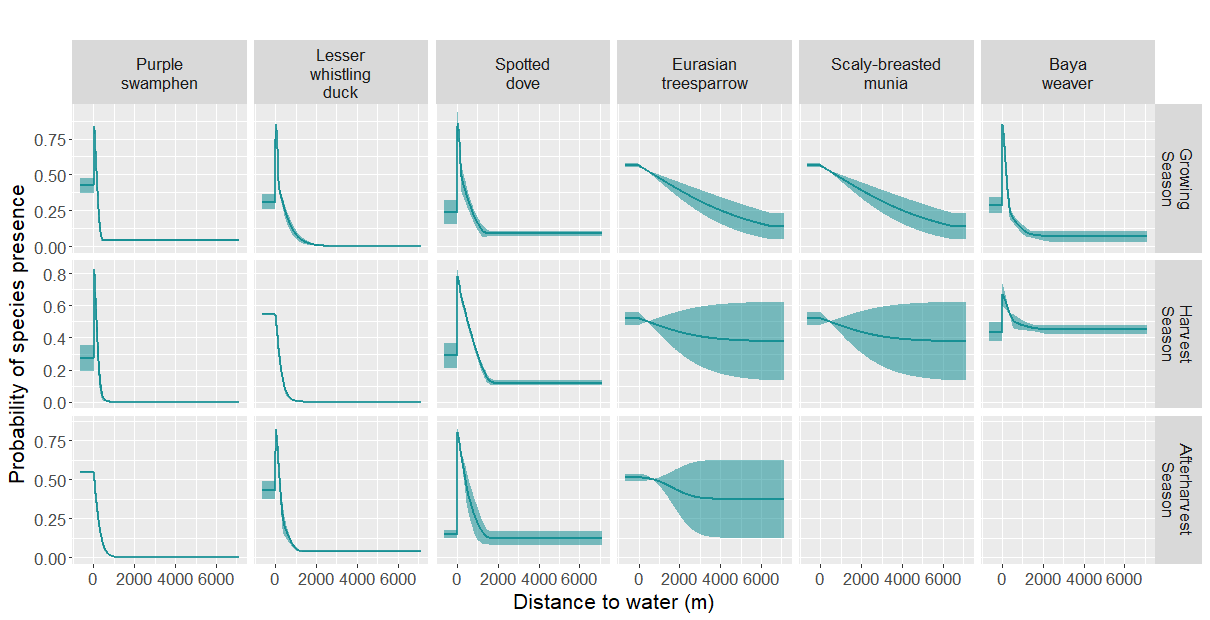


Fig. S8. The effect of distance to water on the present probability of the six bird species that caused serious crop damage


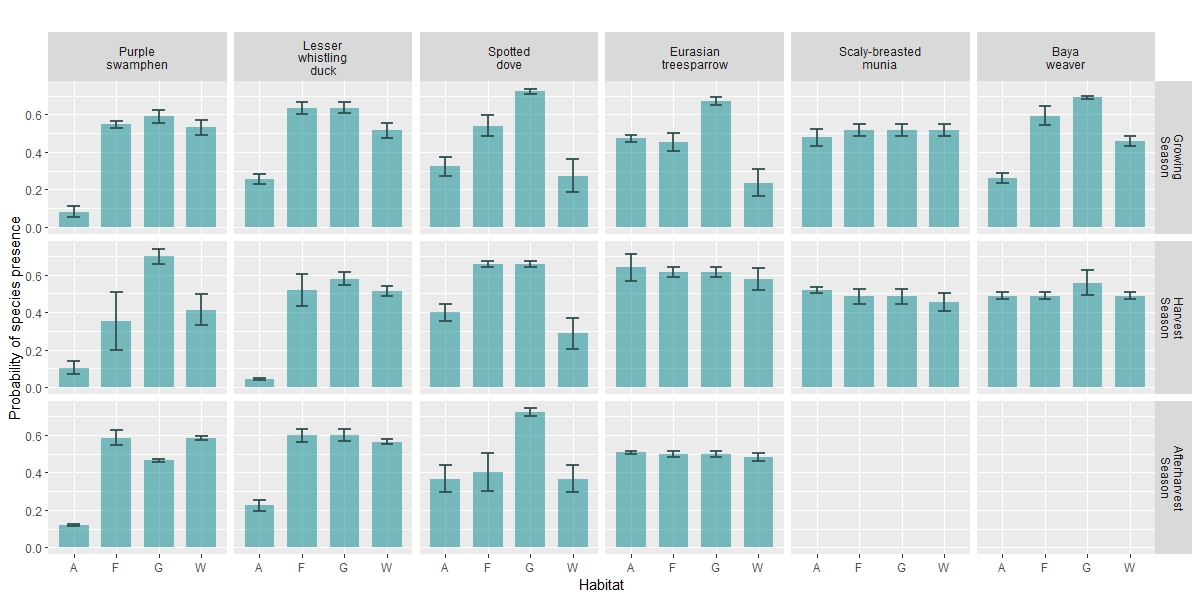


Fig.S9. The effect of habitat factor on the present probability of the six bird species that caused serious crop damage


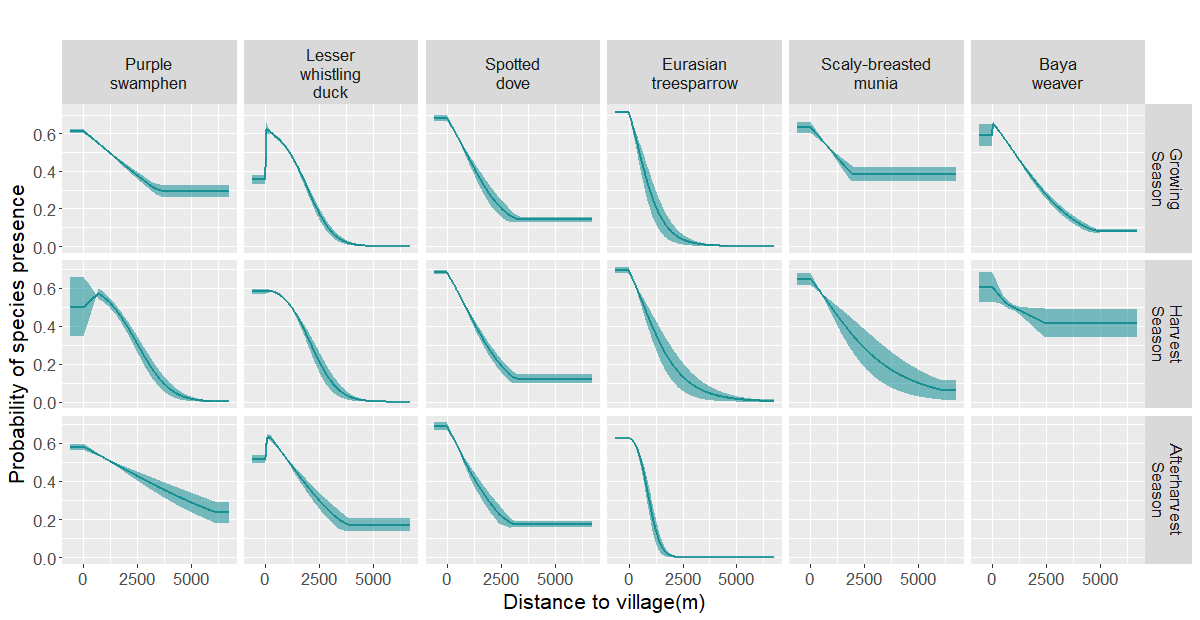


Fig.S10. The effect of distance to village on the present probability of the six bird species that caused serious crop damage


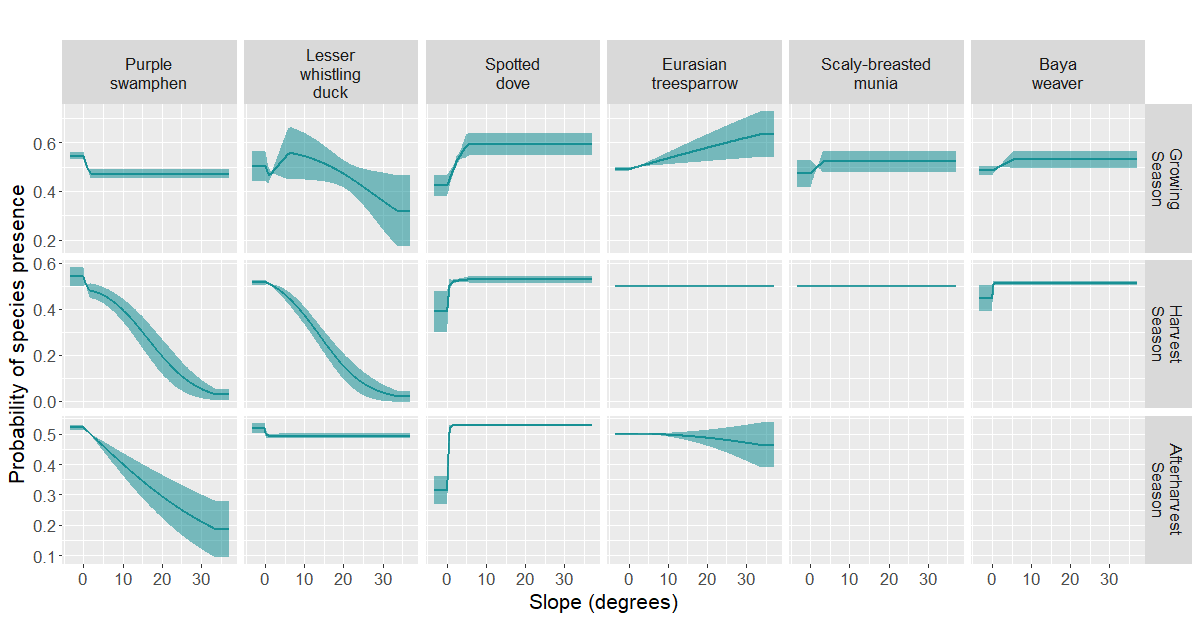


Fig.S11. The effect of slope on the present probability of the six bird species that caused serious crop damage


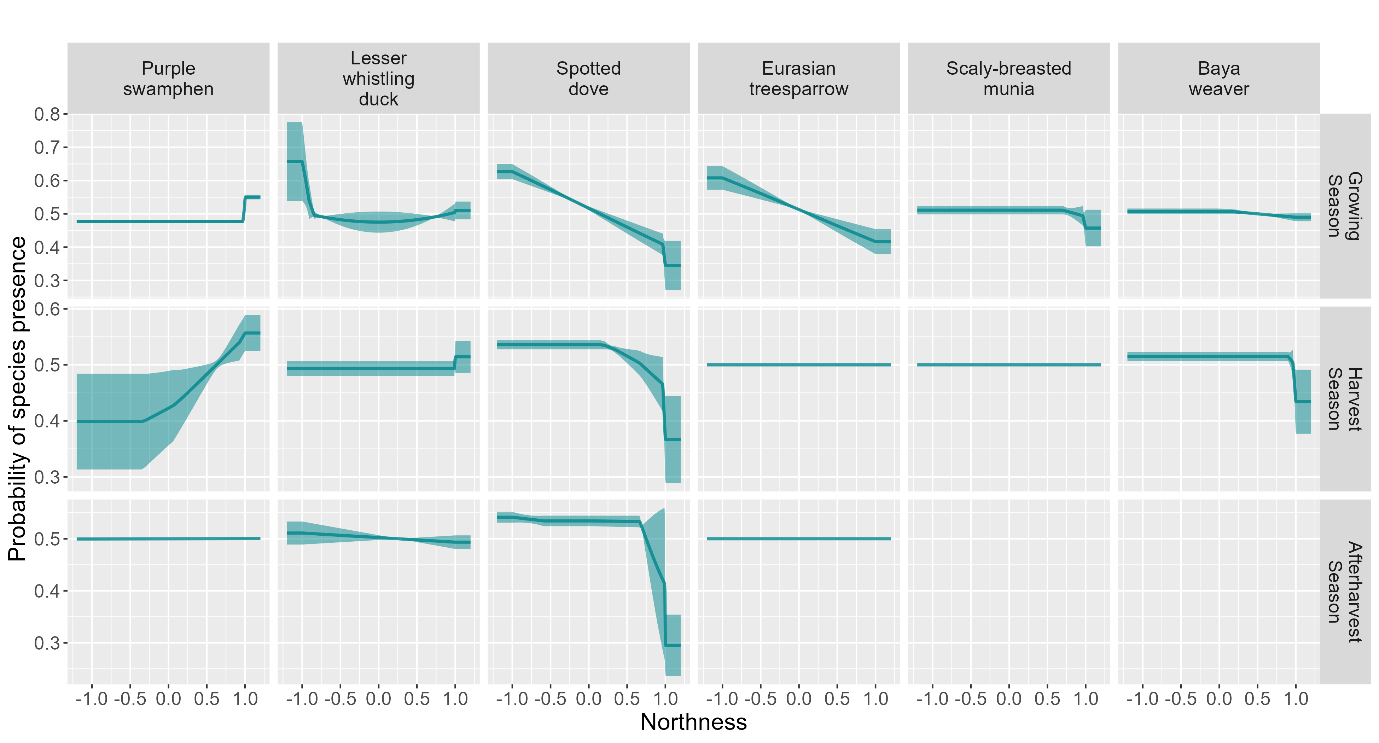


Fig.S12. The effect of northness on the present probability of the six bird species that caused serious crop damage


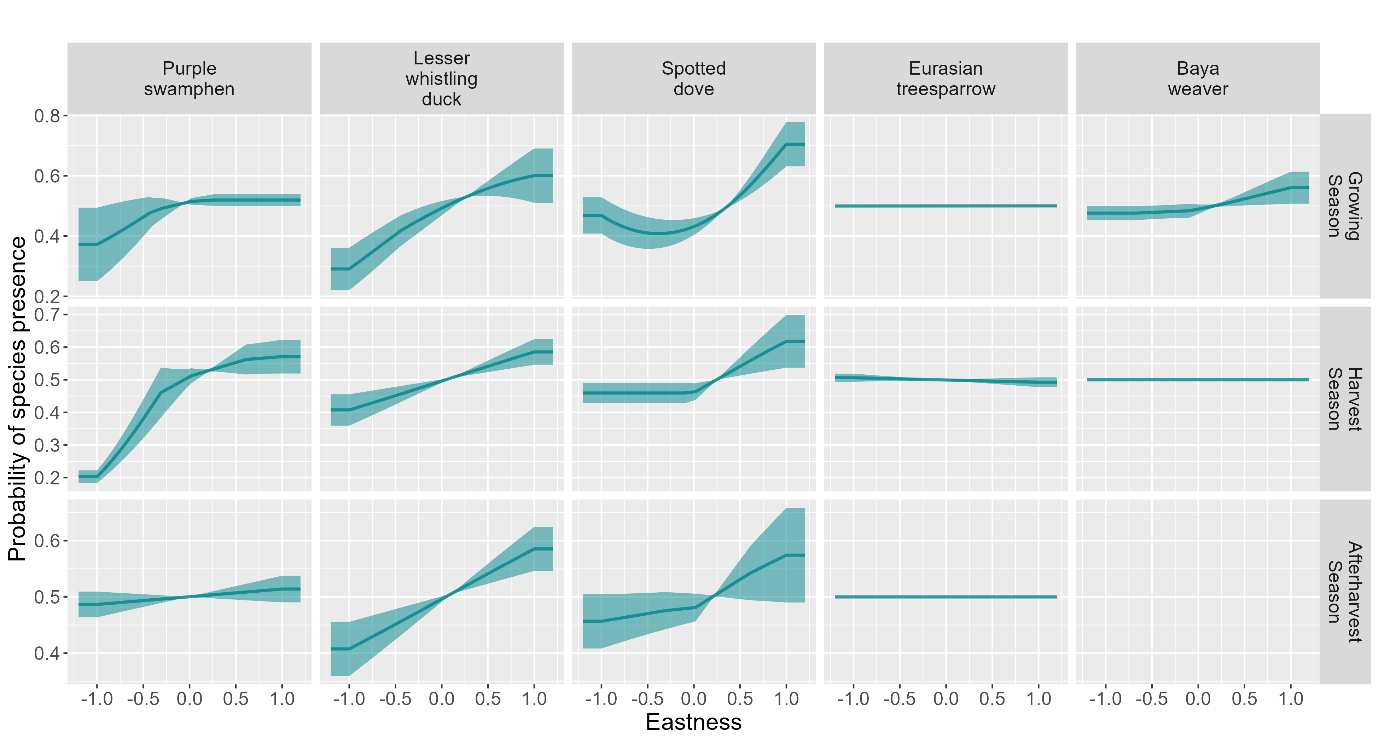


Fig.S13. The effect of eastness on the present probability of the six bird species that caused serious crop damage


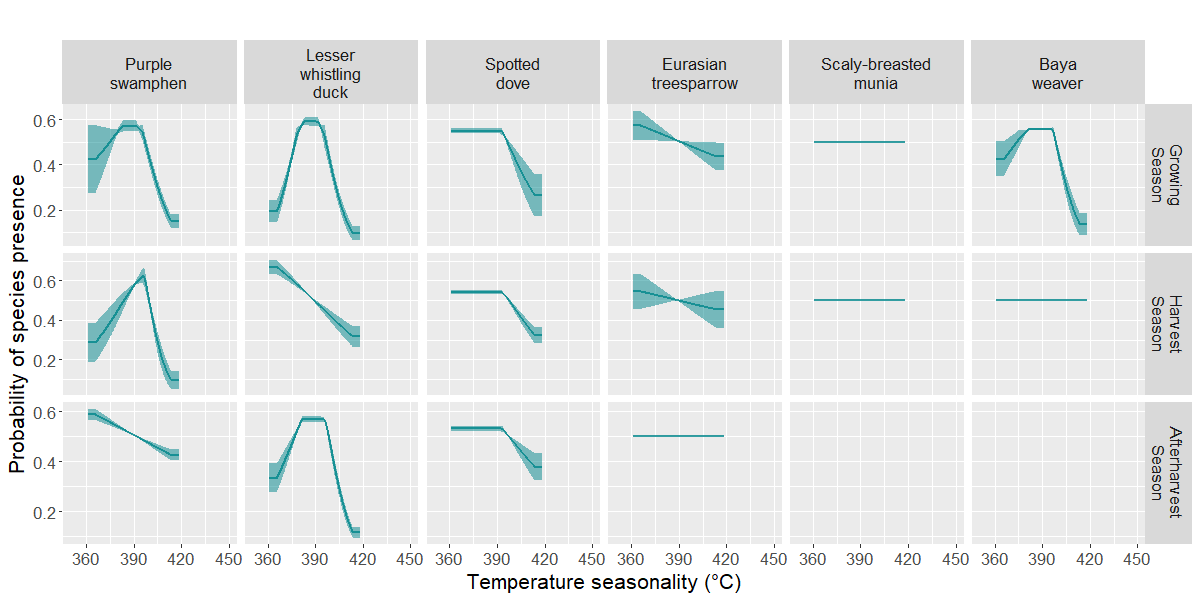


Fig.S14. The effect of temperature seasonality on the present probability of the six bird species that caused serious crop damage


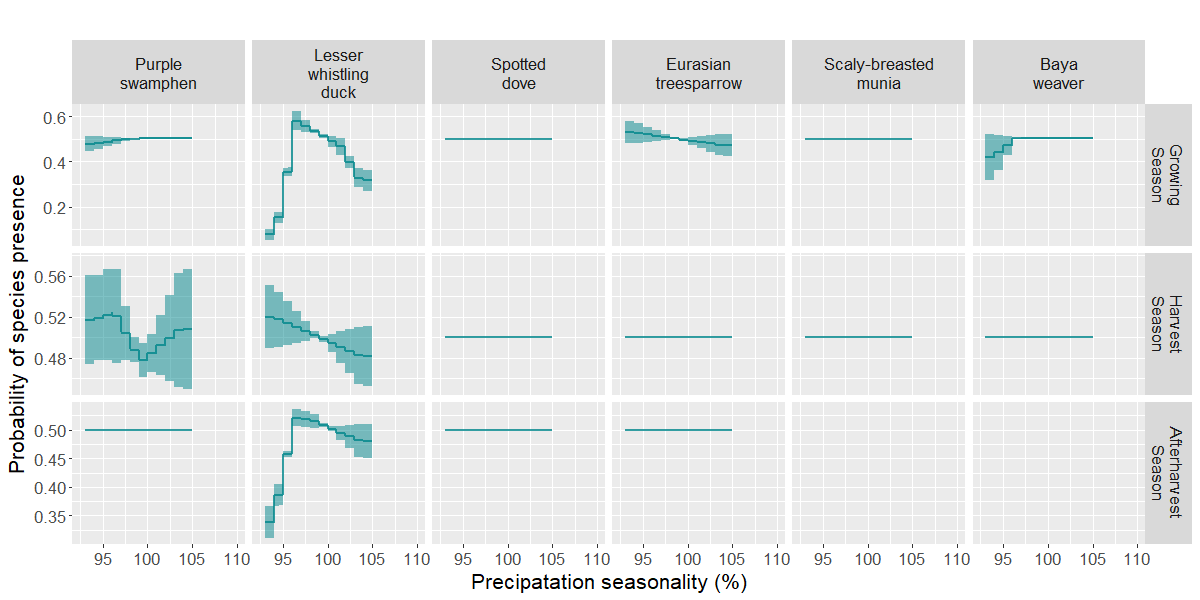


Fig.S15. The effect of precipitation seasonality on the present probability of the six bird species that caused serious crop damage

Table S2. Likelihood ratio test for the importance of environmental predictors on the richness of crop-exploiting bird species in the Indawgyi wetland ecosystem. The importance of each predictor variable is evaluated by comparing the likelihood of full model and the model that reduced a specific predictor. AIC is the Akaike Information Criterion of the model, Df is the degree of freedom for the Chi-square test statistic defined as the difference in number of co(variances) fitted for the full model and reduced model, LRT is the chi-square test statistic for the likelihood-ratio test (i.e., the difference in -2 log-likelihoods between the full model and the reduced model), *P* is the significance of the difference between the two models.

| **Model** | **AIC** | **Df** | **LRT** | ***P*** |
| --- | --- | --- | --- | --- |
| *a.Growing season* |  |  |  |  |
| Full model | 1033.3 | - | - | - |
| **Full model vs Model that reduced habitat** | **1034.0** | **3** | **4.678** | **0.034** |
| Full model vs Model that reduced slope | 1031.3 | 1 | 0.018 | 0.892 |
| Full model vs Model that reduced northness | 1031.8 | 1 | 0.474 | 0.491 |
| Full model vs Model that reduced eastness | 1031.7 | 1 | 0.343 | 0.558 |
| **Full model vs Model that reduced temperature seasonality** | **1036.6** | **1** | **5.308** | **0.021** |
| Full model vs Model that reduced precipitation seasonality | 1032.4 | 1 | 1.082 | 0.298 |
| Full model vs Model that reduced distance to water | 1032.3 | 1 | 0.994 | 0.319 |
| Full model vs Model that reduced distance to village | 1031.4 | 1 | 0.035 | 0.852 |
| *b.Harvest season* |  |  |  |  |
| Full model | 968.06 | - | - | - |
| **Full model vs Model that reduced habitat** | **969.41** | **3** | **7.342** | **0.016** |
| Full model vs Model that reduced slope | 966.13 | 1 | 0.062 | 0.802 |
| Full model vs Model that reduced northness | 967.19 | 1 | 1.125 | 0.289 |
| Full model vs Model that reduced eastness | 968.24 | 1 | 2.180 | 0.139 |
| Full model vs Model that reduced temperature seasonality | 968.38 | 1 | 2.313 | 0.128 |
| Full model vs Model that reduced precipitation seasonality | 966.07 | 1 | 0.010 | 0.920 |
| Full model vs Model that reduced distance to water | 966.97 | 1 | 0.910 | 0.419 |
| Full model vs Model that reduced distance to village | 966.72 | 1 | 0.654 | 0.340 |
| *c.After harvest season* |  |  |  |  |
| Full model | 1000.07 | - |  |  |
| **Full model vs Model that reduced habitat** | **1016.24** | **3** | **22.168** | **<0.001** |
| Full model vs Model that reduced slope | 998.94 | 1 | 0.866 | 0.352 |
| Full model vs Model that reduced northness | 998.45 | 1 | 0.382 | 0.536 |
| Full model vs Model that reduced eastness | 998.87 | 1 | 0.795 | 0.373 |
| Full model vs Model that reduced temperature seasonality | 998.15 | 1 | 0.082 | 0.775 |
| Full model vs Model that reduced precipitation seasonality | 1000.12 | 1 | 2.049 | 0.153 |
| **Full model vs Model that reduced distance to water** | **1005.66** | **1** | **7.584** | **0.005** |
| Full model vs Model that reduced distance to village | 998.08 | 1 | 0.010 | 0.921 |

Table S3. Likelihood ratio test for the importance of environmental predictors on the abundance of crop-exploiting bird species in the Indawgyi wetland ecosystem. The importance of each predictor variable is evaluated by comparing the likelihood of full model and the model that reduced a specific predictor. AIC is the Akaike Information Criterion of the model, Df is the degree of freedom for the Chi-square test statistic defined as the difference in number of co(variances) fitted for the full model and reduced model, LRT is the chi-square test statistic for the likelihood-ratio test (i.e., the difference in -2 log-likelihoods between the full model and the reduced model), *P* is the significance of the difference between the two models.

| **Model** | **AIC** | **Df** | **LRT** | ***P*** |
| --- | --- | --- | --- | --- |
| *a.Growing season* |  |  |  |  |
| Full model | 6185.3 | - | - | - |
| Full model vs Model that reduced habitat | 6180.2 | 3 | 0.891 | 0.827 |
| Full model vs Model that reduced slope | 6183.3 | 1 | 0.018 | 0.891 |
| Full model vs Model that reduced northness | 6183.5 | 1 | 0.152 | 0.696 |
| Full model vs Model that reduced eastness | 6185.3 | 1 | 1.980 | 0.159 |
| **Full model vs Model that reduced temperature seasonality** | **6189.5** | **1** | **6.154** | **0.013** |
| Full model vs Model that reduced precipitation seasonality | 6184.7 | 1 | 1.394 | 0.238 |
| Full model vs Model that reduced distance to water | 6183.5 | 1 | 0.174 | 0.676 |
| Full model vs Model that reduced distance to village | 6183.3 | 1 | 0.001 | 0.976 |
| *b.Harvest season* |  |  |  |  |
| Full model | 3249.0 | - | - | - |
| **Full model vs Model that reduced habitat** | **3252.0** | **3** | **9.067** | **0.028** |
| Full model vs Model that reduced slope | 3248.6 | 1 | 1.644 | 0.199 |
| Full model vs Model that reduced northness | 3247.0 | 1 | 0.006 | 0.939 |
| Full model vs Model that reduced eastness | 3248.3 | 1 | 1.330 | 0.248 |
| Full model vs Model that reduced temperature seasonality | 3247.0 | 1 | 0.035 | 0.851 |
| Full model vs Model that reduced precipitation seasonality | 3247.0 | 1 | 0.005 | 0.946 |
| Full model vs Model that reduced distance to water | 3247.6 | 1 | 0.596 | 0.440 |
| Full model vs Model that reduced distance to village | 3248.1 | 1 | 1.146 | 0.284 |
| *c.After harvest season* |  |  |  |  |
| Full model | 4201.3 | - | - | **-** |
| **Full model vs Model that reduced habitat** | **4213.2** | **3** | **17.908** | **<0.001** |
| Full model vs Model that reduced slope | 4199.6 | 1 | 0.254 | 0.614 |
| Full model vs Model that reduced northness | 4200.1 | 1 | 0.813 | 0.367 |
| Full model vs Model that reduced eastness | 4201.0 | 1 | 1.648 | 0.199 |
| Full model vs Model that reduced temperature seasonality | 4199.3 | 1 | 0.006 | 0.937 |
| **Full model vs Model that reduced precipitation seasonality** | **4206.0** | **1** | **6.702** | **0.009** |
| **Full model vs Model that reduced distance to water** | **4204.2** | **1** | **4.916** | **0.026** |
| Full model vs Model that reduced distance to village | 4199.3 | 1 | 0.011 | 0.917 |

Table S4. Likelihood ratio test for the importance of environmental predictors on the richness of crop-exploiting bird species in the agricultural habitat of the Indawgyi wetland ecosystem. The importance of each predictor variable is evaluated by comparing the likelihood of full model and the model that reduced a specific predictor. AIC is the Akaike Information Criterion of the model, Df is the degree of freedom for the Chi-square test statistic defined as the difference in number of co(variances) fitted for the full model and reduced model, LRT is the chi-square test statistic for the likelihood-ratio test (i.e., the difference in -2 log-likelihoods between the full model and the reduced model), *P* is the significance of the difference between the two models.

| **Model** | **AIC** | **Df** | **LRT** | ***P*** |
| --- | --- | --- | --- | --- |
| *a.Growing season* |  |  |  |  |
| Full model | 262.54 | - | - | - |
| **Full model vs Model that reduced crop stage** | **263.64** | **3** | **7.104** | **0.038** |
| Full model vs Model that reduced slope | 261.50 | 1 | 0.959 | 0.327 |
| Full model vs Model that reduced northness | 260.64 | 1 | 0.101 | 0.750 |
| Full model vs Model that reduced eastness | 261.94 | 1 | 1.404 | 0.236 |
| Full model vs Model that reduced temperature seasonality | 261.11 | 1 | 0.569 | 0.451 |
| Full model vs Model that reduced precipitation seasonality | 260.93 | 1 | 0.392 | 0.532 |
| Full model vs Model that reduced distance to water | 261.19 | 1 | 0.648 | 0.421 |
| Full model vs Model that reduced distance to village | 263.12 | 1 | 2.585 | 0.108 |
| *b.Harvest season* |  |  |  |  |
| Full model | 252.21 | - | - | - |
| Full model vs Model that reduced crop stage | 249.20 | 3 | 2.989 | 0.393 |
| Full model vs Model that reduced slope | 250.21 | 1 | 0.000 | 0.996 |
| Full model vs Model that reduced northness | 254.11 | 1 | 1.896 | 0.084 |
| Full model vs Model that reduced eastness | 250.23 | 1 | 0.021 | 0.883 |
| Full model vs Model that reduced temperature seasonality | 251.82 | 1 | 1.612 | 0.204 |
| Full model vs Model that reduced precipitation seasonality | 253.64 | 1 | 3.427 | 0.064 |
| Full model vs Model that reduced distance to water | 250.22 | 1 | 0.008 | 0.930 |
| Full model vs Model that reduced distance to village | 250.26 | 1 | 0.044 | 0.832 |
| *c.After harvest season* |  |  |  |  |
| Full model | 242.55 | - |  |  |
| Full model vs Model that reduced crop stage | 238.19 | 4 | 3.644 | 0.456 |
| Full model vs Model that reduced slope | 240.70 | 1 | 0.153 | 0.695 |
| Full model vs Model that reduced northness | 244.07 | 1 | 3.528 | 0.060 |
| **Full model vs Model that reduced eastness** | **245.21** | **1** | **4.661** | **0.030** |
| Full model vs Model that reduced temperature seasonality | 240.56 | 1 | 0.012 | 0.911 |
| Full model vs Model that reduced precipitation seasonality | 243.05 | 1 | 2.503 | 0.114 |
| **Full model vs Model that reduced distance to water** | **247.84** | **1** | **7.291** | **0.006** |
| Full model vs Model that reduced distance to village | 240.57 | 1 | 0.023 | 0.877 |

Table S5. Likelihood ratio test for the importance of environmental predictors on the abundance of crop-exploiting bird species in the agricultural habitat of the Indawgyi wetland ecosystem. The importance of each predictor variable is evaluated by comparing the likelihood of full model and the model that reduced a specific predictor. AIC is the Akaike Information Criterion of the model, Df is the degree of freedom for the Chi-square test statistic defined as the difference in number of co(variances) fitted for the full model and reduced model, LRT is the chi-square test statistic for the likelihood-ratio test (i.e., the difference in -2 log-likelihoods between the full model and the reduced model), *P* is the significance of the difference between the two models.

| **Model** | **AIC** | **Df** | **LRT** | ***P*** |
| --- | --- | --- | --- | --- |
| *a.Growing season* |  |  |  |  |
| Full model | 557.28 | - | - | - |
| **Full model vs Model that reduced crop stage** | 562.49 | 3 | 11.205 | **0.010** |
| Full model vs Model that reduced slope | 555.28 | **1** | **0.001** | 0.970 |
| Full model vs Model that reduced northness | **555.37** | 1 | 0.085 | **0.770** |
| **Full model vs Model that reduced eastness** | **559.05** | **1** | **3.771** | **0.025** |
| Full model vs Model that reduced temperature seasonality | 555.28 | 1 | 0.000 | 0.995 |
| **Full model vs Model that reduced precipitation seasonality** | **561.02** | 1 | **5.744** | **0.017** |
| Full model vs Model that reduced distance to water | 556.23 | **1** | 0.950 | 0.329 |
| Full model vs Model that reduced distance to village | 555.75 | 1 | 0.465 | 0.495 |
| *b.Harvest season* |  |  |  |  |
| Full model | 457.53 | - | - | - |
| Full model vs Model that reduced crop stage | 454.91 | 3 | 3.382 | 0.336 |
| Full model vs Model that reduced slope | 456.63 | 1 | 1.106 | 0.292 |
| Full model vs Model that reduced northness | 458.14 | 1 | 2.619 | 0.105 |
| Full model vs Model that reduced eastness | 456.24 | 1 | 0.714 | 0.398 |
| Full model vs Model that reduced temperature seasonality | 455.87 | 1 | 0.341 | 0.559 |
| **Full model vs Model that reduced precipitation seasonality** | **460.26** | **1** | **4.735** | **0.029** |
| Full model vs Model that reduced distance to water | 455.53 | 1 | 0.000 | 0.984 |
| Full model vs Model that reduced distance to village | 456.15 | 1 | 0.623 | 0.429 |
| *c.After harvest season* |  |  |  |  |
| Full model | 602.87 | - | - | - |
| **Full model vs Model that reduced crop stage** | **618.93** | **4** | **24.058** | **<0.001** |
| Full model vs Model that reduced slope | 600.90 | 1 | 0.028 | 0.868 |
| Full model vs Model that reduced northness | 602.03 | 1 | 1.157 | 0.282 |
| **Full model vs Model that reduced eastness** | **605.18** |  | **4.306** | **0.037** |
| Full model vs Model that reduced temperature seasonality | 601.03 | 1 | 0.161 | 0.688 |
| Full model vs Model that reduced precipitation seasonality | 601.74 | 1 | 0.869 | 0.351 |
| Full model vs Model that reduced distance to water | 603.99 | 1 | 3.121 | 0.077 |
| Full model vs Model that reduced distance to village | 601.02 | 1 | 0.153 | 0.695 |

Table S6. Test of spatial autocorrelation using Moran I in the residuals of the models fitted to examine the spatio-temporal distribution of crop-exploiting avian species at the landscape level and at the agricultural habitat level

|  | **Landscape level** | | **Agricultural habitat level** | |
| --- | --- | --- | --- | --- |
| **Season** | **Richness** | **Abundance** | **Richness** | **Abundance** |
| Growing | Moran I = -0.011  *p* = 0.513 | Moran I = -0.805  *p* = 0.999 | Moran I = -0.028  *p* = 0.533 | Moran I = -0.103  *p* = 0.932 |
| Harvest | Moran I = 0.450  *p* = 0.326 | Moran I = -0.775  *p* = 0.999 | Moran I = 0.113  *p* = 0.013 | Moran I = -0.505  *p* = 0.999 |
| After harvest | Moran I = 0.060  *p* = 0.167 | Moran I = -0.639  *p* = 0.999 | Moran I = -0.056  *p* = 0.687 | Moran I =-0.250  *p* = 0.999 |
